# Gut microbiota and innate immune barrier dysfunction in hepatic portal venous gas: a hypothesis-generating review

**DOI:** 10.3389/fimmu.2026.1974872

**Published:** 2026-10-05

**Authors:** Xiaoshuai Bai, Lin Zhu, Yibo Sun, Yanfei Shi, Ping Zhou, Che Wang, Lianxiang Cui, Lei Shi

**Affiliations:** 1 Department of Emergency Internal Medicine, the Affiliated Hospital of Qingdao University, Qingdao, China; 2 Complaint and Petition Office, Qingdao Traditional Chinese Medicine Hospital, Qingdao Department of Oncology, Qingdao Hiser Hospital Affiliated of Qingdao University, Qingdao, China; 3 School of the First Clinical Medicine, Shandong Second Medical University, Weifang, Shandong, China; 4 Department of Environmental Health, Qingdao Municipal Center for Disease Control and Prevention, Qingdao Institute of Preventive Medicine, Qingdao, Shandong, China; 5 Department of Oncology, Qingdao Central Hospital, University of Health and Rehabilitation Sciences (Qingdao Central Hospital), Qingdao, China; 6 Department of Emergency Internal Medicine, Qingdao Hiser Hospital Affiliated of Qingdao University, Qingdao, China; 7 Department of Emergency Internal Medicine, Huangdao District People’s Hospital, Qingdao, China

**Keywords:** bacterial translocation, fecal microbiota transplantation, gut microbiome dysbiosis, gut-liver axis, hepatic immune tolerance, hepatic portal venous gas (HPVG), mucosal immunity, TLR4

## Abstract

Hepatic portal venous gas (HPVG) is an uncommon radiographic sign whose prognosis is driven by bowel necrosis and shock rather than by intraportal gas. Immunological events along the gut–liver axis have not been synthesized for adult HPVG, and dedicated microbiome, metabolomic, gut–vascular barrier (GVB), and immune-profiling data are lacking. This hypothesis-generating review proposes gut dysbiosis as a putative contributor to mucosal and hepatic innate immune failure, inferred from cirrhosis, porto-sinusoidal vascular disease (PSVD), and intestinal ischemia/reperfusion. In those proxy states, loss of acetyl-CoA butyrate-production capacity, context-dependent bile-acid shifts, lipopolysaccharide–Toll-like receptor 4 priming, facultative-anaerobe expansion, and fermentative H_2_/CO_2_ pathways documented in cultured HPVG isolates—Clostridium perfringens (pyruvate–ferredoxin oxidoreductase–hydrogenase) and Escherichia coli (pyruvate-formate lyase–formate hydrogen lyase)—compromise epithelial and GVB integrity. These links are not assembled as a demonstrated sequence in adult HPVG. We propose a physical-core model in which a route of entry and a local driving pressure gradient are necessary, whereas pathway-defined fermentative isolates, dysbiosis, mucosal inflammation, ischemia, and impaired portal clearance are optional amplifiers. Iatrogenic or purely pressure-related HPVG can occur without dysbiosis or a substantial pathogen-associated molecular pattern load; in necrosis-driven HPVG, dysbiosis is proposed only as a concurrent immune amplifier; when HPVG occurs in cirrhosis or PSVD, portal hypertension is treated as a host modifier of barrier geometry and washout, not as an independent generator of intraportal gas. Portal vein thrombosis, portal hypertension, pneumatosis intestinalis, pyogenic liver abscess, and HPVG may share the portal vein as a conduit but are not interchangeable. No microbiota-directed intervention has been tested against an HPVG endpoint and none should delay source control; probiotics, rifaximin, fecal microbiota transplantation, diet, and phage remain research questions.

## 1 Introduction

Hepatic portal venous gas (HPVG) represents a rare radiologic sign whose detection on computed tomography (CT) or ultrasonography often prompts urgent clinical evaluation because the same image may accompany self-limited mucosal injury or bowel necrosis with shock (1, 2). The condition was first radiographically demonstrated in infants by Wolfe and Evans in 1955 and has since been recognized across all age groups (3). HPVG is uncommon, and incidence estimates are approximate: they derive from small single-center series rather than denominator-based population epidemiology. In two academic retrospective reviews of CT examinations, HPVG was identified in 17 of 14,000 scans (0.12%) at one center and in 11 of 19,000 scans (0.06%) at another; later syntheses therefore cite an order of 0.06–0.12% among scanned hospital populations rather than a true population incidence (4, 5). Detection is higher among high-risk groups such as patients with cirrhosis, inflammatory bowel disease (IBD), or recent gastrointestinal surgery (4, 5). The reported detection rate has increased with the wider use and improved resolution of multidetector CT, shifting HPVG from a near-uniformly fatal radiographic curiosity toward a finding with a broad prognostic spectrum (4, 5). Prognosis is heavily etiology-dependent: when HPVG accompanies bowel necrosis or infarction, reported mortality is high—historically as high as 75% in that necrosis-conditioned subset, and greater still in patients with shock or with concurrent pneumatosis intestinalis, which stratifies ischemic risk but is not the same lesion as intraportal gas—whereas benign causes (mucosal disruption without ischemia, iatrogenic pneumatosis) frequently resolve with conservative measures such as bowel rest, antibiotics, and fluid resuscitation (5–7). HPVG can occur without pneumatosis intestinalis, and pneumatosis intestinalis can occur without HPVG. Large emergency-department cohorts confirm that clinical and laboratory markers of bowel ischemia, rather than the mere presence of portal gas, drive immediate outcome, reinforcing the need to understand the biological events that precede gas entry (7). Those triage markers identify hypoperfusion risk. They do not equate bowel ischemia with transmural necrosis, and they do not equate either finding with the proportion of patients who undergo laparotomy. Contemporary all-cause in-hospital mortality for CT-detected HPVG is lower than the historical necrosis-conditioned estimate and was 27.3% in a 1,590-patient Japanese inpatient analysis (8). HPVG should therefore be interpreted as a radiographic marker of an underlying process, not as an independent high-mortality diagnosis; mortality tracks bowel necrosis, shock, and uncontrolled intra-abdominal infection rather than intraportal gas itself.

Etiologic classification traditionally emphasizes mechanical or vascular insults such as mesenteric arterial or venous occlusion, alongside infectious gas production by Clostridium species and other anaerobes, mucosal barrier breaches in IBD or diverticulitis, and iatrogenic factors including endoscopy, surgery, and chemotherapy (4, 5). Recent case series and reviews have linked HPVG to a broad spectrum of precipitating conditions, including emphysematous gastritis and severe neurocritical illness after intracranial procedures. Experimental brain death itself induces intestinal microbiota dysbiosis and hepatic metabolomic derangement, illustrating how systemic critical illness can rapidly reshape the gut–liver axis (9). Mechanistic analyses indicate that HPVG can arise by at least two non-exclusive routes: mechanical entry of ordinary luminal or insufflated gas across a pressure-driven mucosal defect, and local production or tracking of gas by organisms that encode hydrogenogenic fermentation pathways. Despite extensive literature on imaging grading systems and operative-versus-conservative decision algorithms, the molecular and microbial events that precede gas entry have not been mapped by dedicated multi-omics in adult HPVG. The post-2020 expansion of 16S, shotgun metagenomic, metabolomic, and barrier-immune profiling has refined the gut–liver axis in proxy conditions, including cirrhosis and portal hypertension (10, 11), PSVD (12–14), intestinal ischemia/reperfusion (15–17), and necrotizing enterocolitis, rather than in radiographically confirmed adult HPVG. Contemporary reviews and case series from 2024–2025 (4, 5) emphasize that cultured isolates of *Clostridium perfringens* and *Escherichia coli*, which ferment carbohydrate to H_2_ and CO_2_, generate large volumes of gas that can enter the portal system in the setting of ischemia, necrosis, mechanical injury, or perforation. The same literature also documents HPVG after endoscopy, balloon dilation, colonoscopy, contrast or saline enema, vomiting-induced gastric dilatation, ileus, and other pressure-related injuries in which ordinary nitrogen- or carbon dioxide-rich luminal gas may enter without a documented dysbiotic community or a substantial PAMP load. Single-center series continue to identify infection and bowel-wall compromise as recurring themes, although systematic microbiome profiling remains absent. These two routes, which can operate independently, frame the later physical-core model (Section 4.4).

In this review, “gas-forming” is used only in a restricted, organism-level sense. It denotes a cultured isolate whose species encodes a documented hydrogenogenic fermentation pathway capable of generating H_2_ and CO_2_ *in vitro*—pyruvate–ferredoxin oxidoreductase–hydrogenase in *Clostridium perfringens*, pyruvate-formate lyase–formate hydrogen lyase in *Escherichia coli*, and mixed-acid and/or 2,3-butanediol fermentation in Klebsiella spp.—and that has been recovered from blood, tissue, or luminal fluid in radiographically confirmed HPVG (direct clinical for isolation; the enzymes themselves are species-level biochemistry). The term does not mean that gas was demonstrated in the clinical microbiology laboratory for that isolate. It does not mean that the patient had an emphysematous infection, such as gas gangrene, emphysematous gastritis, a gas-forming pyogenic liver abscess, or emphysematous cystitis. It does not mean that intramural gas (pneumatosis intestinalis) was present. It does not mean that the lucencies on CT have been chemically identified as microbial H_2_ or CO_2_ rather than luminal or insufflated N_2_ or CO_2_. The molecular composition of HPVG gas has not been measured. Isolation of a gas-forming organism is therefore evidence for a bacterial route of entry or of local production, not proof that microbial gas is the radiographic species. Emphysematous tissue gas, intramural gas, and portal venous gas occupy successive anatomic compartments and are not interchangeable terms. Gas within a Klebsiella liver abscess, hepatic venous gas after abscess rupture, and septic portal-vein thrombophlebitis share the portal vein as a conduit with HPVG but are not equivalent to bowel-derived HPVG. The same non-substitution rule applies to portal vein thrombosis and to portal hypertension: neither is a gaseous manifestation, and neither is an HPVG equivalent.

Non-interchangeable portal-axis entities. Portal vein thrombosis, including septic pylephlebitis, portal hypertension, pneumatosis intestinalis, pyogenic liver abscess, and HPVG may be reported together because they occupy neighboring stations on the portal axis. They are not interchangeable vascular or infectious manifestations. Pneumatosis intestinalis is intramural gas within the bowel wall. It can serve as a transit compartment between the lumen and the mesenteric veins, but it is not required for HPVG, it is not a laboratory definition of a gas-forming isolate, and hydrogen sampled from pneumatosis-cystoides cysts is not a chemical identification of portal gas. Iatrogenic, vomiting-related, and isolated pressure-related HPVG frequently occur without mural gas. Portal hypertension is a hemodynamic host state defined by an elevated portosystemic gradient, not by intraportal lucencies. It can change route geometry through splanchnic congestion, mucosal edema, and gut–vascular barrier (GVB) failure, and it can slow or redistribute a finite gas bolus after entry. Raised downstream mesenteric or portal venous pressure is not hypothesized to generate intraportal gas, and HPVG in a patient who already has portal hypertension is recorded as a host covariate rather than as a validated third HPVG phenotype. Portal vein thrombosis and pylephlebitis are occlusive diseases of the portal or mesenteric lumen. In pylephlebitis series, an infectious gastrointestinal source and thrombus predominate, whereas portal gas is an inconstant accompanying sign and is incidental to the clot. A null association between stool microbiota and portal vein thrombosis therefore cannot adjudicate an HPVG mechanism. Pyogenic liver abscess, including gas-forming and hypervirulent *Klebsiella pneumoniae* abscess, is a parenchymal infection. Intracavitary abscess gas, hepatic venous gas after abscess rupture, and septic portal-vein thrombophlebitis share a conduit with HPVG but are not bowel-derived HPVG. Hypervirulence loci explain post-translocation survival in abscess biology; they do not encode hydrogenogenic fermentation and have not been demonstrated in radiographically confirmed adult HPVG. Throughout this review, coexistence on imaging never licenses substitution of one of these terms for another.

The gut–liver axis provides a biologically plausible, mechanistically tractable framework. **Figure 1** separates a sufficient iatrogenic or pressure-related route of ordinary luminal or insufflated N_2_/CO_2_ (direct clinical) from an optional dysbiosis-amplifier route in selected non-iatrogenic settings (hypothesis when applied to HPVG). The portal vein delivers microbial metabolites, pathogen-associated molecular patterns (PAMPs), and translocated bacteria to the liver, while hepatic bile acids, secretory IgA, and Kupffer-cell activity exert retrograde influence on microbial composition (10, 18–22). This bidirectional anatomy makes the liver both a target of gut-derived signals and a regulator of the luminal environment, so that perturbations at either end propagate around the loop (22, 23). Under stress, antibiotic exposure, high-fat diet, or preexisting liver disease, this equilibrium collapses into dysbiosis—reduced α-diversity, loss of acetyl-CoA butyrate-pathway capacity, and expansion of facultative anaerobes that can respire host-derived electron acceptors and ferment carbohydrate to H_2_ and CO_2_ (10, 20, 24). In cirrhosis and related gut–liver diseases, such dysbiosis is linked to compromised tight junctions that permit luminal microbes and microbial products to enter the portal circulation, lipopolysaccharide-driven TLR4 priming of hepatic innate cells that is associated with inflammation and portal hypertension in those diseases, and a shift from obligate-anaerobic butyrate fermentation toward net accumulation of H_2_, CH_4_, and other VOCs when hydrogenogenic mixed-acid or clostridial fermentation outpaces hydrogenotrophic methanogenesis, sulfate reduction, or acetogenesis (4, 11, 25). These associations are indirect clinical findings in proxy gut–liver diseases and are not assembled here as a linear TLR4–inflammasome–NET mechanism of HPVG. Reverse causality is equally important: cirrhosis and portal hypertension themselves reshape the microbiome via altered bile-acid pools, reduced motility, and splanchnic congestion (26, 27). Genetic-epidemiologic and cohort-plus-colonization analyses offer additional, if indirect, support for taxa-specific effects in liver disease, though such inferences warrant cautious interpretation (19, 28). Whether any of these pathways produces radiographic intraportal gas is examined as hypothesis in Section 4.

**Figure 1:**
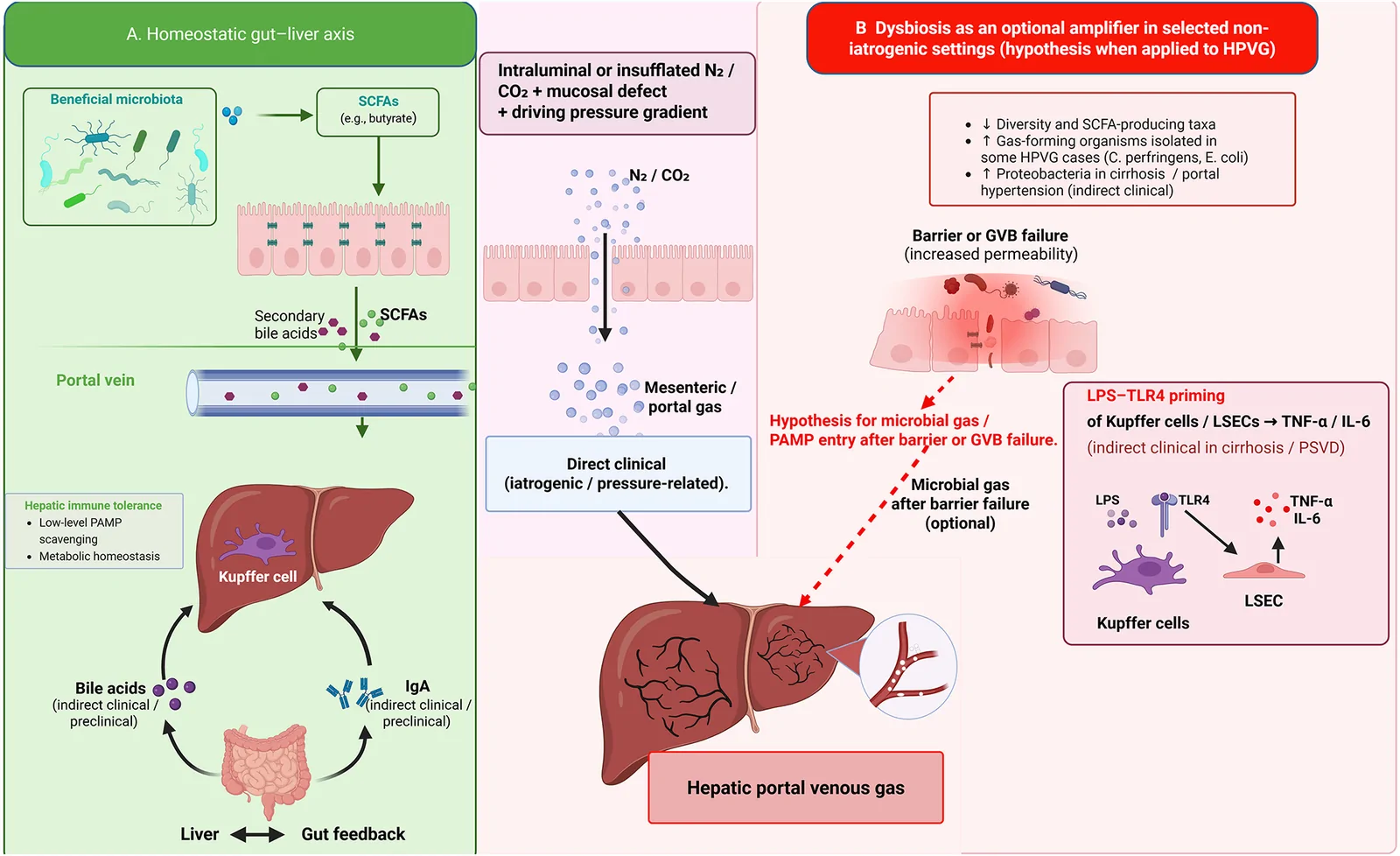
Homeostatic gut–liver axis and two non-equivalent routes to hepatic portal venous gas. **(A)** Homeostatic circuitry: SCFA- and bile-acid-supported barrier tone and hepatic immune tolerance (indirect clinical and preclinical in health and in proxy gut–liver disease). This panel is not a mechanism that prevents portal gas. Centre: ordinary luminal or insufflated N_2_/CO_2_ plus a mucosal defect and a driving pressure gradient is sufficient for iatrogenic or purely pressure-related HPVG (direct clinical) and does not require community-level dysbiosis or a substantial PAMP load. **(B)** Dysbiosis as an optional amplifier in selected non-iatrogenic settings (hypothesis when applied to HPVG). Reduced diversity and acetyl-CoA butyrate-production capacity, pathway-defined fermentative isolates recovered in some HPVG cases (C. perfringens, E. coli; direct clinical for isolation) read by fermentation pathway (PFOR–hydrogenase; PFL–FHL), facultative-anaerobe expansion in cirrhosis or portal hypertension (indirect clinical), barrier or GVB failure, and LPS–TLR4 priming of Kupffer cells and LSECs with TNF-α/IL-6 release (indirect clinical in cirrhosis and PSVD) are shown as optional modifiers. “Gas-forming” in this panel means isolate-level pathway capacity. It does not mean emphysematous infection, pneumatosis, or chemically identified portal gas. The dashed arrow marks hypothesized microbial-gas or PAMP entry after barrier failure. Solid arrows indicate processes shown in HPVG or in related human disease. The image is not a treatment algorithm. Created in BioRender. Bai, X. (2026) https://BioRender.com/a54lxgs.

Direct microbiome evidence in HPVG remains limited by the condition’s rarity, ethical constraints on invasive sampling in acutely ill patients, and a historical focus on macroscopic vascular findings. To our knowledge, no dedicated adult HPVG cohort with 16S rRNA sequencing, shotgun metagenomics, metabolomics, GVB readouts, or mucosal immunophenotyping has been published. Post-2020 multi-omics therefore informs this review only through proxy diseases, not through an HPVG index cohort. Most mechanistic insight is therefore extrapolated from proxy states—cirrhosis with portal hypertension, porto-sinusoidal vascular disease (PSVD), a recently formalized non-cirrhotic portal-hypertensive entity with microvascular lesions, and ischemic or inflammatory bowel pathology (29, 30). A 2025 narrative review positions gut dysbiosis as an under-researched crossroads in PSVD, citing increased permeability, microbial translocation, and downstream effects on liver sinusoidal endothelial cells (LSECs), hepatic stellate cells (HSCs), and Kupffer cells (12–14). Parallel syntheses of the gut–liver axis in portal hypertension reach convergent conclusions, identifying gut-derived endotoxin and metabolite dysregulation as shared drivers across cirrhotic and non-cirrhotic disease (31–33). Those conclusions apply to portal hypertension and microvascular liver injury (indirect clinical). They supply the proxy substrate for Section 4; they do not themselves constitute an HPVG mechanism.

The clinical stakes of this biology are concrete. The central management decision in HPVG, immediate surgical exploration versus a trial of conservative therapy, still rests largely on imaging pattern and gross clinical status, tools that discriminate poorly in the intermediate-risk patient who is neither clearly septic-necrotic nor obviously benign (4). Because the microbial and barrier events reviewed here precede and shape the transition from reversible mucosal injury to irreversible transmural necrosis, they are candidate sources of exactly the early, biologically grounded signals that current algorithms lack. A clearly labelled working model is therefore more than narrative synthesis: it is a prerequisite for specifying which biomarkers and interventions are worth testing, and in which HPVG phenotype (developed as proposals in Sections 5 and 6).

This review is distinguished from recent syntheses of cirrhotic portal hypertension or PSVD by three tasks. First, it asks whether proxy gut–liver immune pathways can be organized into an HPVG-specific working model without replacing the two classical, independently sufficient routes of gas entry (Section 4.4). Second, it treats iatrogenic pressure-related HPVG, acute necrosis-driven HPVG, and HPVG that occurs in a patient who already has portal hypertension as different immunological questions rather than as three validated disease subtypes (Section 3.3). Third, it converts that framework into a construct-specific research menu and a validation agenda, while separating research candidates from any clinically qualified triage test (Sections 5–7). Because dedicated adult HPVG multi-omics and immunophenotyping cohorts were not identified, the assembled cause-and-effect chain is inferred from proxy systems and is offered as a set of testable propositions rather than an established mechanism. Literature from 2020 onward is prioritized for proxy multi-omics and for recent HPVG clinical series, not because multi-omics has been applied to adult HPVG. Older landmark papers are retained when they established a classical HPVG mechanism or a core gut–liver construct.

Four descriptive evidence categories, not GRADE ratings, are used in the remainder of the text, in the figures, and in **Table 1**. Each major link is labelled as: (1) direct clinical, demonstrated in patients with HPVG; (2) indirect clinical, demonstrated in a related human condition (cirrhosis, PSVD, IBD, necrotizing enterocolitis, or clinical intestinal ischemia); (3) preclinical, supported only by animal or *in-vitro* experiments; or (4) hypothesis, newly proposed in this article. GRADE certainty ratings are not used, because GRADE is designed for bodies of outcome evidence that inform recommendations, not for individual molecular pathways or schematic arrows. Classical mechanical mucosal disruption with raised luminal or insufflation pressure, iatrogenic HPVG after endoscopy or enema, isolation of gas-forming bacteria in some HPVG cases, and independence of the mechanical and bacterial routes are direct clinical. A sentence that joins several links inherits the weakest category, so the assembled dysbiosis-to-HPVG sequence is hypothesis. When that assembled model is discussed, we use proposed, may, or could.

**Table 1:** Evidence table: mechanistic links in HPVG.

Mechanistic link	Population and design	Endpoint	Indirectness	Label
HPVG is a radiographic sign with etiology-dependent mortality	Adult and pediatric case series and clinical reviews	Imaging diagnosis; death; need for surgery	1	Direct clinical
Ordinary luminal or insufflated nitrogen- and carbon dioxide-rich gas can enter through a pressure-driven mucosal defect without community-level dysbiosis, a dominant gas-forming pathobiont, or a substantial PAMP load	HPVG after endoscopy, balloon dilation, colonoscopy, barium or saline enema, vomiting-induced gastric emphysema, and isolated bowel distension	Portal gas with conservative resolution in the absence of ischemia	1–2	Direct clinical
Bacterial route: gas-forming organisms isolated from blood or tissue in some HPVG cases	HPVG case series and autopsy	Culture of *Escherichia coli*, *Clostridium perfringens*, Klebsiella, *Citrobacter*, *Candida*	1	Direct clinical
Combined mechanical and bacterial entry	Autopsy with pneumatosis, HPVG, and enteric bacteria in blood and liver	Tissue gas plus cultured gas-formers	1–2	Direct clinical
Community-level stool or mucosal dysbiosis in adult HPVG	None identified	16S or shotgun composition	4	Hypothesis
Metabolomic, GVB/PV-1, or immune-profiling studies in adult HPVG	None identified	SCFA, PV-1, cytokines, single-cell maps	4	Hypothesis
Reduced α-diversity, loss of acetyl-CoA butyrate-production capacity, facultative-anaerobe expansion with mixed-acid or 2,3-butanediol fermentation potential	Human cirrhosis and portal hypertension, cross-sectional 16S and reviews	Composition and inferred pathway capacity; LPS; HVPG; SBP; HE. Associations are qualitative or study-specific; no pooled diversity odds ratio and no universal LPS–HVPG coefficient are claimed.	2	Indirect clinical
Shared LPS-producing genera in stool and portal blood	Human cirrhosis with portal-vein thrombosis; paired feces and portal blood 16S	Shared taxa; portal LPS	2	Indirect clinical
Elevated LPS, and a higher commercial zonulin-family peptide ELISA signal, in PSVD, including without clinical portal hypertension.	Human PSVD case–control plus histology	LPS; zonulin-family peptide ELISA (not authentic pre-haptoglobin-2; not a dual-sugar leak index); endothelial and platelet activation.	2	Indirect clinical
Circulating PV-1 as a GVB surrogate across cirrhosis stages	Human stage-stratified cirrhosis; research ELISA, not a standardized clinical assay	PV-1; EndoCAb; LBP; survival	2	Indirect clinical in cirrhosis; circulating PV-1 is not an IVD and has not been measured in HPVG
NEC or severe IBD with dysbiosis, pneumatosis, and portal gas	Neonatal NEC and IBD series	Clinical co-occurrence	2	Indirect clinical
Microbiota tunes intestinal ischemia–reperfusion injury and NETosis	Germ-free, antibiotic-depleted, and I/R mice	Mucosal injury; NETs; permeability	3	Preclinical
Cirrhosis, not isolated prehepatic portal hypertension, disrupts GVB	Bile-duct ligation and CCl4 versus partial portal-vein ligation	PV-1; FITC-dextran leak; GFP-E. coli to liver	3	Preclinical
hvKp immune evasion and gut-to-liver translocation	Human liver-abscess series; mouse translocation models	Abscess; virulence genes	2–3	Indirect clinical and preclinical
Physical core (route of entry plus favorable pressure or flow gradient) is necessary; gas-forming organisms, dysbiosis, inflammation, ischemia, GVB failure, and impaired clearance are optional amplifiers rather than universal co-requisites	Conceptual synthesis in this article, constrained by iatrogenic and cadaver mechanical evidence	Not measured	4 for the amplifier layer; 1 for independence of the mechanical route	Direct clinical for independent mechanical entry; hypothesis for the optional-modifier layer; elevated portal pressure is not treated as a facilitator of entry
In non-iatrogenic HPVG, radiographic gas burden is hypothesized to be higher when high fermentative output coexists with GVB leak; this is a testable prediction rather than a validated equation and does not apply to isolated insufflation of air or carbon dioxide	Conceptual synthesis in this article	Not measured	4	Hypothesis
Acute necrotic HPVG: dysbiosis as amplifier; HPVG in a chronic portal-hypertensive host: dysbiosis as an untested upstream modifier of barrier failure and clearance, not a validated phenotype	Clinical series for etiology fractions; immunology from cirrhosis and PSVD proxies; no dedicated cirrhosis- or PSVD-restricted HPVG cohort	Necrosis versus chronic PH as host context	2–4	Direct clinical for etiology mix; hypothesis for immune roles; hypothesis/evidence gap for an independent PH effect
Probiotics or synbiotics reduce HVPG or HE recurrence	Randomized trials and meta-analyses in cirrhosis	HVPG; HE	2	Indirect clinical
FMT is safe in stable cirrhosis with prior overt HE; a *post hoc* contrast found lower HE recurrence (THEMATIC). This is not an HPVG result.	Phase II randomized trial in cirrhosis with prior overt HE	HE recurrence; safety	2–3	Indirect clinical
Unselected probiotics in predicted severe pancreatitis increase ischemia and death	PROPATRIA randomized trial	Bowel ischemia; mortality	2	Indirect clinical
Phage or next-generation probiotics for gas-forming pathobionts, proposed only as preclinical tools for future defined-consortium experiments, not as HPVG therapy.	Mouse liver-disease models; no HPVG trial	Liver injury in animals	3–4	Preclinical and hypothesis
Barrier, injury, translocation, and fermentation research markers plus breath hydrogen and methane tested, after necrosis exclusion, as covariates of subsequent course in a research protocol, not as tools that assign surgery versus conservative care versus microbiota-directed treatment.	Individual constructs studied in cirrhosis; no component is a qualified HPVG diagnostic; commercial zonulin ELISA, circulating PV-1, LBP/sCD14, and blood 16S each carry the assay limits in **Table 3**. Commercial zonulin-family peptide kits are not treated as paracellular-leak assays.	Not tested	4	Hypothesis

MEDLINE/PubMed was searched from inception through 10 September 2026, and the reference lists of key papers were hand-searched. Search terms combined HPVG descriptors (“hepatic portal venous gas”, “portal venous gas”, “portomesenteric venous gas”, HPVG) with gut–liver and immunity terms (dysbiosis, microbiome, microbiota, gut–liver axis, bacterial translocation, TLR4, short-chain fatty acid, fecal microbiota transplantation) and proxy conditions (cirrhosis, portal hypertension, PSVD, intestinal ischemia, necrotizing enterocolitis, inflammatory bowel disease); targeted searches were added for *Clostridium perfringens*, *Escherichia coli*, *Klebsiella pneumoniae*, and *K. oxytoca*. A supplementary search for hypervirulent *Klebsiella pneumoniae* was used only to determine whether genotypically defined hvKp has been isolated from radiographically confirmed adult HPVG; no such isolate was identified. Human studies from 2020 onward were prioritized for recent HPVG clinical series and for multi-omics in proxy gut–liver diseases; this date cut-off does not imply that an adult HPVG multi-omics cohort exists. Older landmark papers were retained when they established a classical HPVG mechanism or a core gut–liver construct. Sources were included if they addressed HPVG itself, barrier or innate-immune data in proxy diseases, microbiota-directed interventions with immune or portal-hemodynamic endpoints, or transferable animal or *in-vitro* pathways; isolated imaging quizzes were excluded. A study was treated as HPVG-specific only when HPVG was radiographically confirmed; all other human diseases were treated as indirect clinical sources. When clinical series reported etiology fractions, bowel or mesenteric ischemia, transmural necrosis or infarction, and operative or histopathological findings were extracted as separate endpoints and were not pooled. No adult HPVG cohort with 16S sequencing, shotgun metagenomics, metabolomics, GVB/PV-1 measurement, or mucosal immunophenotyping was identified. Positive and null findings on the same claim were both retained and interpreted by matching population and endpoint. GRADE was not used. Evidence labels follow the four categories defined above; short codes (D-HPVG, D-proxy, A/IV, H) refer to those categories.

## 2 Homeostatic immune circuitry of the gut–liver axis

### 2.1 Microbial community structure as an immune setpoint

Under physiological conditions, the intestinal microbiome exists in symbiosis with the host. High-rank taxonomic dominance by Bacillota (Firmicutes) and Bacteroidota, with smaller contributions from Actinobacteriota, Pseudomonadota, and Verrucomicrobiota, is a frequent *but* geographically and method-dependent background pattern; it is not used here as an immune setpoint (34–36). A fixed healthy setpoint of approximately 50% Bacteroidetes and 40% Firmicutes is not used here. Phylum-level shares vary with geography, diet, age, early-life immune education, socioeconomic context, and sequencing pipeline, and they do not define health or HPVG risk. Community structure is not merely taxonomic. It contributes to the antigenic and metabolic setpoint of mucosal and hepatic immunity. Healthy adults often show comparatively high α-diversity, although Shannon values are platform-dependent (sequencing method, rarefaction depth, and taxonomic rank) and no single numeric cutoff is validated as a health threshold or as an HPVG risk metric (36, 37). Rather than a single healthy configuration, human gut communities occupy a range of compositional states that nonetheless converge on conserved immunoregulatory outputs, including acetyl-CoA butyrate-production capacity, succinate- or propanediol-dependent propionate formation, bile-acid 7α-dehydroxylation by *bai*-positive specialists, and tonic stimulation of pattern-recognition receptors below the threshold that triggers overt inflammation (22, 38). Functional redundancy, meaning that multiple taxa perform overlapping metabolic and immunostimulatory tasks, confers resilience, so that moderate perturbations need not collapse barrier immunity or hepatic tolerance. It is the erosion of this redundancy, rather than the loss of any single species or any phylum ratio, that most reliably marks the transition from a tolerant to a pro-inflammatory community, a principle now formalized in composite microbiome-health indices (37). In liver disease, longitudinal work shows that diversity loss and the expansion of oral-type taxa and facultative anaerobes capable of nitrate or oxygen respiration track disease stage and coincide with rising translocation of microbial ligands, providing the compositional backdrop against which the innate immune events below unfold (23, 38). These associations are supported by observational data linking higher microbial diversity to reduced bacterial translocation and to adverse liver-disease trajectories in cirrhosis and related cohorts. No reconstructable primary analysis was identified that reports an odds ratio of approximately 0.7 for diversity and liver-disease progression, and that figure is therefore not used here. One example of a sourced, still-indirect estimate is a cirrhosis cohort in which severe dysbiosis independently predicted death over four years (hazard ratio 8.6, 95% confidence interval 1.9–38.0); that endpoint is mortality in cirrhosis, not HPVG, and the confidence interval is wide (indirect clinical; associative, not GRADE-rated). For the present immunological framework, the operative implication is that loss of diversity is a measurable risk state for impaired colonization resistance, heightened innate sensing of lipopolysaccharide and other PAMPs, and erosion of hepatic immune tolerance.

### 2.2 Short-chain fatty acids as epithelial–immune signals

Selected commensal taxa, not gut phyla as blocs, ferment indigestible dietary fibers into short-chain fatty acids, principally butyrate, acetate, and propionate (39–41).Phylum-level abundance cannot reliably predict SCFA output or immune function. Colonic butyrate-production capacity is a pathway phenotype. Butyrate potential is concentrated in a restricted set of Bacillota families, including Lachnospiraceae and Ruminococcaceae or Oscillospiraceae, and is carried mainly by the acetyl-CoA route (thiolase/atoB, β-hydroxybutyryl-CoA dehydrogenase, crotonase, butyryl-CoA dehydrogenase–electron-transfer flavoprotein, and a terminal butyryl-CoA:acetate CoA-transferase [but] or butyrate kinase [*buk*]). Alternative glutamate- and lysine-fed butyrate routes exist but contribute little community-level flux and cannot rescue collapse of the acetyl-CoA pathway. Propionate is enriched in many Bacteroidota via the succinate pathway and in some Bacillota via the propanediol pathway. Acetate is a near-universal fermentative output and is also generated by reductive acetogenesis. Many Firmicutes do not produce butyrate, and pathway presence is more conserved at family or species level than at phylum level. These metabolites are immunologically active. Butyrate serves as the primary energy substrate for colonocytes, promotes mucus secretion, and activates GPR43 (FFAR2) and GPR109A to stimulate epithelial proliferation and upregulate tight-junction proteins including zonula occludens-1 (ZO-1) and claudins (42–44). GPR109A signaling further supports secretory IgA-dependent containment of luminal bacteria. The three principal SCFAs are functionally distinct: acetate, the most abundant, is largely absorbed and reaches peripheral immune tissues; propionate is delivered directly to the liver via the portal vein, where it participates in metabolic and immunomodulatory signaling; and butyrate acts locally to fuel the epithelium and to condition mucosal immune cells (39–41). Through histone-deacetylase inhibition, butyrate reprograms epithelial and immune gene expression toward a barrier-protective, anti-inflammatory phenotype, promotes regulatory T-cell differentiation, and stabilizes hypoxia-inducible factor to reinforce the mucosal oxygen gradient that restrains facultative anaerobes and limits unnecessary innate activation (45). This division of labor means that dysbiosis-driven SCFA loss simultaneously weakens the epithelial barrier, reduces regulatory tone, and alters the metabolite profile presented to Kupffer cells and LSECs. Because of functional redundancy, similar SCFA outputs can arise from taxonomically different communities; the immunologically relevant readout is metabolite supply and pathway completeness, not a Firmicutes-to-Bacteroidetes ratio. A comprehensive 2024 synthesis details how the SCFA–host interface integrates diet, microbiota, and epithelial–immune signaling, and how its collapse recurs across gastrointestinal and hepatic disease (45). These SCFA-dependent immune functions are precisely those whose loss initiates the barrier and innate-sensing failure examined in Section 4. **Figure 2** summarizes these commensal metabolite outputs and their effects on epithelial junctions, Treg/IL-10 tone, and portal LPS load in health and in cirrhosis cohorts (indirect clinical). The circuitry is not a demonstrated defence against intraportal gas and is not an indication for probiotic therapy.

**Figure 2:**
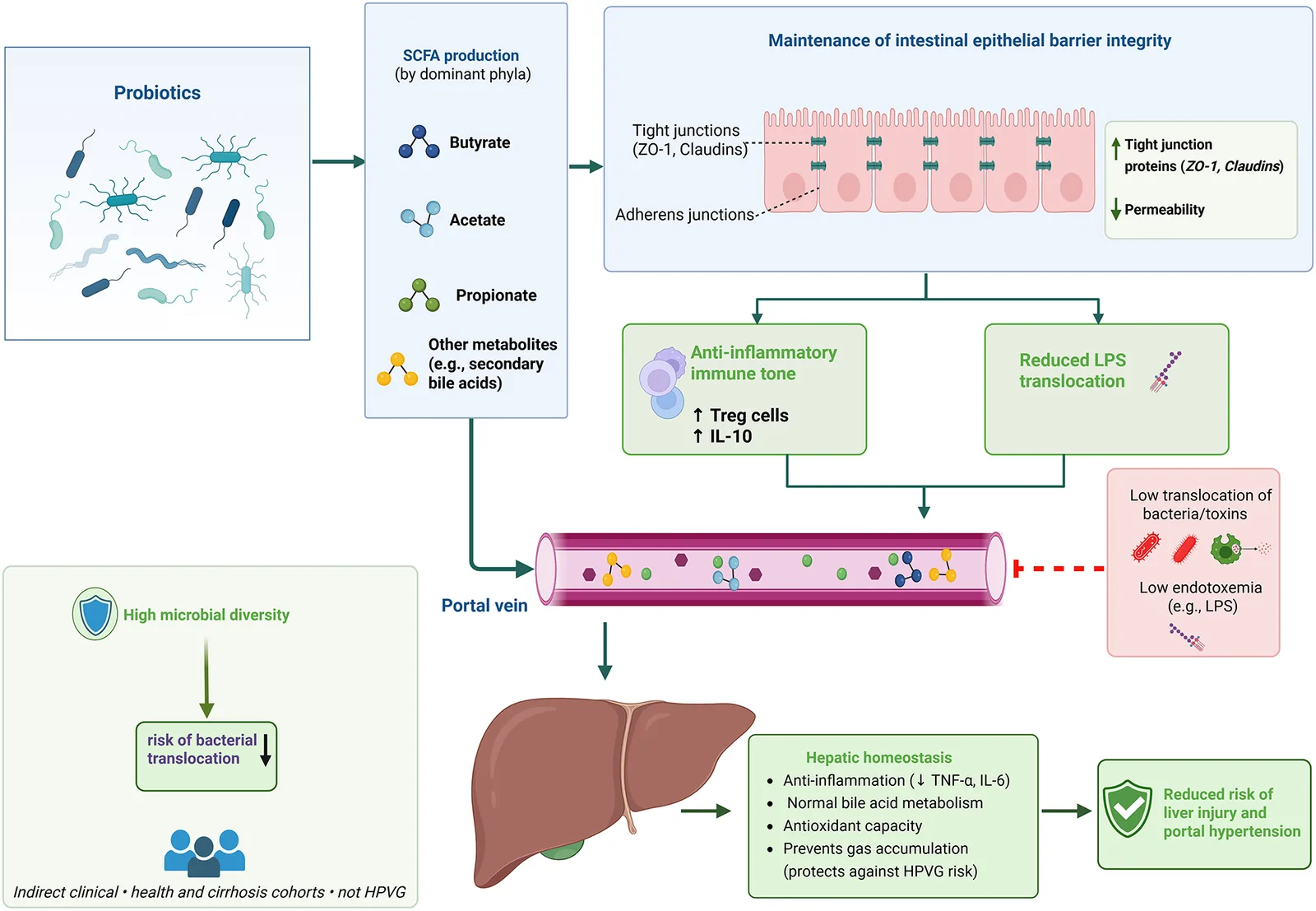
Commensal SCFA and bile-acid outputs that set epithelial and hepatic immune tone in health and in proxy liver disease. Selected commensal taxa generate butyrate, acetate, propionate and context-dependent secondary bile acids that support tight-junction proteins (ZO-1, claudins), raise Treg/IL-10 tone and limit portal LPS. Higher diversity is linked to lower bacterial translocation in health and cirrhosis cohorts (indirect clinical; not measured in HPVG). SCFA production is pathway- and taxon-restricted and is not assigned to dominant phyla. Butyrate is shown as acetyl-CoA pathway capacity; propionate as succinate or propanediol pathway capacity; facultative-anaerobe restraint is shown as a butyrate- and hypoxia-dependent function, not as a Firmicutes-to-Bacteroidetes ratio. This image does not depict prevention of intraportal gas, does not assign an HPVG risk metric, and is not an indication for probiotics or other microbiota-directed products. Created in BioRender. Bai, X. (2026) https://BioRender.com/ls4pab8.

### 2.3 Bile-acid receptors and mucosal immune conditioning

Primary bile acids synthesized in the liver undergo 7α-dehydroxylation by a restricted set of Clostridia that carry the *bai* operon, conventionally placed among Firmicutes and exemplified by Clostridium and Eubacterium species, but not by Firmicutes as a phylum, to generate secondary bile acids such as lithocholic acid and deoxycholic acid (46). These ligands activate the farnesoid X receptor (FXR) and Takeda G-protein-coupled receptor 5 (TGR5) in enterocytes, hepatocytes, and innate immune cells, thereby modulating lipid and glucose metabolism, suppressing hepatic inflammation, and exerting negative feedback on bile-acid synthesis (46–50). The composition of the bile-acid pool is therefore both a readout of microbial function and a determinant of mucosal immune tone. Firmicutes richness is not equated here with a generally protective, secondary-bile-acid-enriched state. At physiologic concentrations, secondary bile acids can support FXR- and TGR5-dependent barrier and metabolic tone. Deoxycholic acid and lithocholic acid are also hydrophobic and can be hepatotoxic, pro-fibrotic, or genotoxic when concentrated or when receptor and conjugation profiles change; other secondary species, including ursodeoxycholic acid and selected oxo- or iso-lithocholic acid metabolites, are often cytoprotective. The sign of the effect is therefore concentration- and context-dependen (47, 48). FXR and TGR5 signaling directly supports epithelial and innate defenses by tightening junctions, inducing antimicrobial peptides, promoting secretory IgA-dependent containment, and restraining inflammasome activation (51). Secondary bile-acid metabolites also modulate RORγ+ regulatory T-cell homeostasis in the gut. The liver in turn releases conjugated bile acids and secretory IgA into the intestine, reinforcing colonization resistance and sustaining beneficial taxa (20, 52). Disruption of this bidirectional bile-acid–immune dialogue, as occurs when 7α-dehydroxylating taxa are depleted, can weaken metabolic, mechanical, and immunological defenses, but a secondary-rich pool is not treated as uniformly beneficial (51).

### 2.4 Hepatic immune tolerance at the portal interface

SCFAs and secondary bile acids promote regulatory T-cell differentiation and interleukin-10 (IL-10) production while limiting pro-inflammatory Th17 responses (50, 53). Kupffer cells and LSECs are continually exposed to low-level microbial products via the portal vein, yet they maintain tolerance through anti-inflammatory signaling, efficient scavenging of PAMPs, and restrained antigen presentation (52, 54). This hepatic firewall normally prevents gut-derived bacteria and PAMPs from reaching the systemic circulation. Its integrity depends on both the quantity of the translocating load and the tolerogenic tone set by microbial metabolites (10, 52). When that tone is lost, the same cells switch from clearance and tolerance to nuclear factor-κB (NF-κB)–driven cytokine release, inflammasome priming, and endothelial activation, which are the proximal immune events linking dysbiosis to portal inflammation. Higher gut microbial diversity correlates with reduced bacterial translocation and slower progression of liver disease (10), and preclinical fecal microbiota transplantation (FMT) from healthy donors into germ-free or dysbiotic animals ameliorates liver injury through SCFA-dependent anti-inflammatory and anti-fibrotic effects (54).

### 2.5 Mucus, antimicrobial peptides, and the GVB as immune checkpoints

Barrier function is not a single membrane but a series of immune defenses arranged in depth, and each layer is microbially conditioned. The outermost defense is the mucus bilayer: a firm inner layer, built chiefly from MUC2 mucin and normally sterile, overlying a looser outer layer colonized by commensals (20). SCFAs and a balanced community sustain mucin synthesis and appropriate mucin foraging, so that dysbiosis, through either mucus thinning or excessive mucin degradation by mucophilic taxa, erodes this first immunological line before the epithelium is breached (20, 45). Beneath the mucus, Paneth and epithelial cells secrete antimicrobial peptides (defensins, Reg3 lectins) and secretory IgA that spatially segregate bacteria from the mucosal surface; secondary bile acids and GPR109A signaling reinforce IgA-dependent containment (44, 50). Deepest lies the GVB, a specialized endothelial–pericyte–enteric-glia unit that gates the passage of bacteria, microbial ligands, and macromolecules from the lamina propria into the portal and systemic circulations (55, 56). The GVB is a distinct immune checkpoint from the epithelial barrier and can fail independently. Its disruption, marked by upregulation of plasmalemma vesicle-associated protein (PV-1), permits viable bacteria and endotoxin to reach the portal vein even when the epithelial layer is only modestly compromised (56). Because the GVB is the last structure separating luminal contents from portal blood, its failure in proxy liver diseases permits viable bacteria and endotoxin to reach the portal vein (indirect clinical). We propose that the same checkpoint could be the anatomic and immunological point at which gut-derived gas, organisms, and PAMPs gain access to the portal venous system in HPVG (hypothesis). Direct GVB or PV-1 measurements in adult HPVG have not been reported.

Therefore, these homeostatic mechanisms create a multilayered immune barrier that prevents pathological translocation of microbial products in health and in proxy gut–liver disease (indirect clinical and preclinical). Loss of this architecture is the starting point of the pathways examined in Section 4. The circuitry itself is established in health and in proxy liver diseases, not in HPVG cohorts.

## 3 Immunological evidence linking dysbiosis to HPVG and its limits

### 3.1 Immune-relevant evidence from portal-hypertensive and ischemic proxy states

Although dedicated microbiome or immunophenotyping studies in HPVG are essentially absent in adults, observational and preclinical evidence from related conditions supports dysbiosis as a biologically plausible upstream modulator of innate immunity in those proxy states (indirect clinical and preclinical). Directly HPVG-specific evidence is confined to case reports and small retrospective series documenting mucosal injury, ischemia, or isolation of gas-forming bacteria (direct clinical) and cannot, on its own, establish an immune or microbiome mechanism. No claim in this section should be read as a completed HPVG pathogenesis. Cross-sectional 16S rRNA studies in cirrhosis and portal hypertension consistently show reduced α-diversity, depletion of taxa that carry acetyl-CoA butyrate-production capacity within Lachnospiraceae and Oscillospiraceae, and expansion of facultative anaerobes, chiefly Enterobacterales, that can respire nitrate or oxygen and perform mixed-acid or 2,3-butanediol fermentation; selected histotoxic Clostridia may co-expand when necrosis is present (10, 11). These functional shifts correlate with elevated circulating lipopolysaccharide (LPS), increased hepatic venous pressure gradient (HVPG), and higher rates of spontaneous bacterial peritonitis (SBP) and hepatic encephalopathy (HE), both of which are translocation-driven immune complications (11, 53). Recent reviews dedicated to cirrhotic portal hypertension converge on the same dysbiosis–endotoxemia–innate-vascular axis and nominate it as a therapeutic target (32, 33). A circulating 16S rRNA signature has additionally been reported in the blood of patients with portal hypertension (57). That finding is compatible with intermittent translocation of microbial DNA, but blood is a low-biomass compartment. Reagent and kit contamination, host-DNA dominance, and the absence of a reproducible core blood microbiome in large healthy cohorts mean that circulating 16S profiles should not be read as straightforward evidence of viable bacterial translocation (58). Culture of gas-forming organisms from blood or tissue in selected radiographically confirmed HPVG cases remains the only direct-clinical microbial evidence in this disease (**Table 2**). In PSVD, a non-cirrhotic portal-hypertensive state, recent work implicates gut-derived endotoxemia and microbial metabolites in LSEC dysfunction, innate endothelial activation, and microvascular thrombosis (12–14).

**Table 2:** Pathway-defined fermentative isolates recovered in radiographically confirmed HPVG, distinguished from hypervirulent *Klebsiella pneumoniae* liver-abscess biology.

Organism or pathotype	Setting in which it has been recovered	What the isolate shows	What it does not show	Evidence category
*Clostridium perfringens*	Ischemic or necrotic bowel; some septic HPVG series	A cultured isolate encoding PFOR–hydrogenase H_2_/CO_2_ fermentation on the bacterial route of HPVG	That community-level dysbiosis was the cause of overgrowth; that the isolate produced gas in the clinical laboratory; that the patient had gas gangrene; that pneumatosis was present; that portal gas was chemically H_2_/CO_2_.	Direct clinical
*Escherichia coli*	Ischemia, intra-abdominal infection, occasional enteritis-associated HPVG	A cultured isolate encoding mixed-acid PFL–FHL H_2_/CO_2_ fermentation on the bacterial route of HPVG	That E. coli is required for iatrogenic or purely pressure-related HPVG; that portal gas was assayed as microbial H_2_/CO_2_.	Direct clinical
*Citrobacter*, *Candida*, mixed anaerobes	Selected septic or necrotic cases	Occasional cultured co-isolates	A dominant or necessary role	Direct clinical, uncommon
Untyped *Klebsiella pneumoniae* or *K. oxytoca*	Case-level HPVG with necrotic colorectal tumor, emphysematous gastritis, or immunosuppressed ileitis	Enterobacterales can ferment carbohydrate to H2 and CO2 by mixed-acid and/or 2,3-butanediol pathways and have been cultured in a minority of HPVG cases	That the isolate was genotypically defined hvKp, or that Klebsiella is a frequent cause of HPVG; that emphysematous gastritis (intramural infection) is the same construct as a fermentative isolate or as HPVG	Direct clinical, case-level
Genotypically defined hvKp (*iucA*, *iroB*, plasmid *rmpA*/*rmpA2*, *peg-344*)	Community-acquired pyogenic liver abscess and metastatic infection; gut-to-liver translocation models	Viable organisms can travel from gut to liver in the portal vein and evade innate clearance via *iucA*, *iroB*, *rmpA*/*rmpA2*, and *peg-344*; these loci do not encode gas formation	That hvKp is a frequent cause of HPVG, that hypervirulence determinants generate portal gas, or that liver-abscess gas equals bowel-derived HPVG	Indirect clinical and preclinical for liver abscess; not demonstrated in HPVG

In this article, “gas-forming” refers only to column 3: a cultured isolate with a documented hydrogenogenic fermentation pathway recovered in radiographically confirmed HPVG (construct A). It does not refer to gas detected in a Durham tube or other laboratory headspace assay for that clinical isolate, to emphysematous infection of tissue (construct B), to intramural gas or pneumatosis intestinalis (construct C), or to chemical identification of the gas that is visible in the portal vein (construct D). Constructs B, C, and D may coexist with a gas-forming isolate but are not implied by the label.Portal hypertension is a hemodynamic host state and is not a construct in this table. Portal vein thrombosis and pylephlebitis are occlusive vascular diseases and are not constructs in this table. Pyogenic liver abscess, including gas-forming or hypervirulent Klebsiella abscess, is a parenchymal infection listed only to mark a related conduit, not an HPVG equivalent.

Animal models add mechanistic support. Antibiotic-induced dysbiosis in rodents elevates intestinal permeability, portal LPS, and portal pressure, and cirrhosis models exhibit dysbiosis preceding overt portal hypertension (52).Human cohort data further link dysbiotic signatures and circulating endotoxin to portal-pressure elevation and adverse outcomes (14). A distinct but instructive immunological proxy is acute intestinal ischemia/reperfusion (I/R), the pathophysiology most directly shared with necrosis-driven HPVG. Germ-free and antibiotic-depletion experiments show that the resident microbiota shapes the severity of I/R injury and that commensal depletion can attenuate some forms of mucosal damage (15–17). In acute mesenteric I/R, resident commensals restrict NETosis while supporting neutrophil recruitment; germ-free or antibiotic-depleted mice show exaggerated LPS-triggered NET formation, an effect that can be dampened by colonization or by enteric LPS. That finding describes microbial priming of neutrophils in an I/R model. It does not show that community-level dysbiosis of the cirrhosis type unleashes NET-mediated HPVG, and NET biology has not been measured in adult HPVG (preclinical). Specific taxa, including *Bifidobacterium bifidum*, mitigate I/R-associated barrier failure in preclinical models (59). These data establish that microbial composition is an active modifier of neutrophil recruitment and NETosis after an ischemic insult (preclinical). This is the closest experimental analogue to necrosis-driven HPVG. Conservative management of benign or infection-associated HPVG, in which microbial and innate immune factors are increasingly implicated, is also being reported with growing frequency (4).

A second instructive proxy is the group of primary intestinal inflammatory disorders in which pneumatosis and portal venous gas themselves occur: IBD, necrotizing enterocolitis, and severe enterocolitis. These conditions couple overt dysbiosis with mucosal immune injury, ulceration, and gas-forming overgrowth, illustrating one human setting in which an immune barrier breach and a microbial gas source coincide. IBD cohorts show characteristic loss of diversity and depletion of SCFA producers alongside barrier dysfunction and innate mucosal activation (35). Neonatal necrotizing enterocolitis, historically the paradigmatic cause of infantile portal venous gas, is now understood as a dysbiosis-driven disease in which Proteobacterial blooms precede pneumatosis and inflammatory necrosis.IBD-associated HPVG is nevertheless frequently iatrogenic: colonoscopy, biopsy, or barium enema can drive ordinary insufflated gas across already ulcerated mucosa, showing that a microbial gas source is not required even when the mucosa is inflamed (direct clinical). Although these entities differ from adult ischemic HPVG in their triggers, they provide the clearest available human illustration that dysbiosis, mucosal innate activation, and portal gas can co-occur in the same patient (indirect clinical). Co-occurrence supports biological plausibility. It does not prove that the same sequence operates in adult HPVG, and it does not make a microbial gas source a requirement for every adult event.

### 3.2 Bidirectional causality between dysbiosis and hepatic immune activation

The gut–liver immune relationship is bidirectional. Dysbiosis can drive hepatic innate activation, while established liver disease and portal hypertension reshape the microbiome through altered bile-acid pools, reduced motility, and splanchnic congestion (26). The same taxonomic appearance, namely expansion of facultative anaerobes and oral-type Bacilli with depletion of acetyl-CoA butyrate-pathway taxa within Clostridia, can therefore be generated by at least three non-identical processes that must not be collapsed into a single HPVG sequence. Arrow A is a community shift that precedes and aggravates intrahepatic vascular resistance in proxy portal hypertension. Arrow B is splanchnic congestion, hypomotility, and an altered bile-acid pool that secondarily reshape an already colonized lumen. Arrow C is acute ecological collapse from antibiotics, mucosal ischemia, fasting, or critical illness, which can mimic chronic dysbiosis within hours to days. Cross-sectional 16S profiles cannot assign a given adult with radiographic HPVG to arrow A, B, or C.

A 2025 cross-sectional analysis of cirrhotic and non-cirrhotic portal hypertension further suggests that portal hypertension itself, rather than reduced hepatic synthetic function, may be the dominant driver of the characteristic cirrhosis-associated dysbiosis pattern, namely expansion of Proteobacteria and Bacilli with depletion of Clostridia. In that study, patients with extrahepatic portal obstruction after portal vein thrombosis, cirrhosis without portal vein thrombosis, and cirrhosis with chronic portal vein thrombosis differed from healthy controls in similar directions and did not differ significantly from one another in beta-diversity, which favors arrow B as a sufficient producer of the cirrhosis-like stool pattern (indirect clinical). This raises the possibility that many microbial signatures previously attributed to cirrhosis are secondary to elevated portal pressure and the immune milieu that accompanies it (60). That contrast did not measure the hepatic venous pressure gradient, bile-acid speciation, or barrier histology, and it was not an HPVG cohort. Mendelian-randomization and cohort analyses have been interpreted as supporting taxa-specific effects, for example Prevotella-linked signals, in liver-disease progression, but such inferences remain indirect and hypothesis-refining rather than confirmatory (28). Transfer of stool from patients with cirrhotic portal hypertension, or monocolonization with a cirrhotic-portal-hypertension-associated *Veillonella* isolate, can aggravate experimental cirrhotic portal hypertension, which supports a modulatory role for selected taxa on portal hemodynamics in that proxy disease and does not demonstrate generation of intraportal gas (indirect clinical and preclinical).

Conversely, recent data in PSVD demonstrate elevated circulating LPS and a higher commercial zonulin-family peptide ELISA signal even in patients without clinically evident portal hypertension, implying that gut-barrier failure and endotoxemia can precede and potentially contribute to the microvascular and innate endothelial lesions that later generate portal hypertension. In this review that ELISA signal is read only as commercial zonulin-family peptide immunoreactivity, not as authentic zonulin/pre-haptoglobin-2 and not as a quantitative index of paracellular leak; dual-sugar permeability testing and tissue PV-1 were not reported in those series (13). Preclinical contrasts point in the same direction: bile-duct ligation or carbon tetrachloride models that combine parenchymal injury, cholestasis, and portal hypertension disrupt the GVB, whereas isolated partial portal-vein ligation, which raises pressure with relative preservation of the hepatic parenchyma, produces a weaker GVB phenotype (preclinical). Elevated downstream mesenteric or portal pressure is therefore not treated as an independent generator of intraportal gas.

Disentangling arrow A from arrow B in human cohorts requires a comparative rather than a single-arm design. A minimum matrix separates pressure from parenchymal failure: (i) cirrhosis with clinically significant portal hypertension, ideally with a measured hepatic venous pressure gradient or portal-vein velocity; (ii) non-cirrhotic extrahepatic portal obstruction or PSVD with portal hypertension; (iii) PSVD or early liver disease without clinically significant portal hypertension; and (iv) non-diseased controls. Concordant cirrhosis-like dysbiosis in arms (i) and (ii) favors arrow B. Elevated translocation or barrier-adjacent signals in arm (iii) favor a step that can precede a measurable portosystemic gradient. A post-hepatic congestion arm, for example Budd–Chiari syndrome, would further test whether any rise in portal pressure is sufficient or whether bile-acid and intrahepatic immune context are required. None of these arms has been completed in radiographically confirmed adult HPVG.

Temporal order requires paired sampling around a pressure-changing event and, separately, around the HPVG index image. Transjugular intrahepatic portosystemic shunt series in cirrhosis show only partial and endpoint-heterogeneous community shifts after a reduction in the portosystemic gradient, including enrichment of selected autochthonous taxa or a fall in fecal proinflammatory medium-chain fatty acids in some cohorts, without a consistent recovery of alpha-diversity (101) (indirect clinical). Those studies were powered for HE, sarcopenia, or metabolomic change, not for intraportal gas, and shunt creation also alters bile-acid cycling, diet, and concomitant drugs. They therefore supply a template for a pressure-reduction sampling frame, not an HPVG result. Bile-acid speciation should be recorded in parallel with taxonomy, because 7α-dehydroxylating capacity can move with cholestasis independently of the portosystemic gradient (26). In an HPVG protocol the index CT must be timestamped against stool or mucosal sampling, and a recovery window after withdrawal of antibiotics, fasting, and decompression is required before any sample is treated as a pre-morbid community.

The most informative designs therefore remain longitudinal studies that capture the temporal sequence of microbial, immunological, and hemodynamic change, and interventional studies in which deliberate microbiome correction is followed by measurement of barrier biomarkers, cytokine profiles, portal pressure, or, only as an exploratory imaging readout, gas burden. No such interventional endpoint has been measured in HPVG. The mechanistic weight of the present review therefore rests on the experimentally supported barrier, translocation, and innate-sensing pathways detailed in Section 4. Directionality between portal hypertension and community composition is an open controversy in proxy disease. When that controversy is applied to HPVG it is hypothesis. It must be resolved before microbiota-directed immunomodulation can be confidently prioritized in any non-necrotic research window, and it does not license classification of dysbiosis as an upstream driver of intraportal gas.

### 3.3 Acute necrotic HPVG versus HPVG in a chronic portal-hypertensive host: amplifier versus untested host modifier

This subsection does not treat dysbiosis as an upstream driver of intraportal gas in a chronic portal-hypertensive host. The two roles distinguished here are a proposed concurrent immune amplifier in necrosis-driven HPVG and an untested host modifier of barrier geometry and post-entry washout when portal hypertension is already present. The predominantly chronic proxy populations must be explicitly contrasted with the acute nature of many HPVG cases. Bowel ischemia, transmural bowel necrosis, and operative or histopathological findings are related but not equivalent endpoints, and published HPVG percentages cannot be pooled across them. In CT-era series, mesenteric or bowel ischemia as the attributed underlying disease typically occupies the 40–53% range: 43% in the Kinoshita cumulative review (7), 40% in a 30-patient cohort that defined ischemia by combined clinical and intraoperative findings (61), and 53% (842 of 1,590) in a Japanese national inpatient analysis (8). Transmural necrosis is a stricter and smaller subset. Wei et al. listed intestinal necrosis as an etiology in 7 of 21 patients (33.3%), but peritonitis, recent abdominal surgery, necrosis, and gastrointestinal bleeding were not mutually exclusive labels in that series (4). Ji et al. reported a combined “intestinal ischemia/necrosis” category in 14 of 25 patients (56%) on the basis of history and CT rather than uniform histopathology; 11 of those 14 patients underwent emergency resection, so tissue-confirmed necrosis was at most 44% of that cohort and was not separately itemized (62). Koizumi et al. keep the two constructs apart: 53% of patients had bowel ischemia, yet only 32.2% of that ischemic subgroup underwent surgery (8). Laparotomy itself is a management decision, not a biological surrogate for necrosis. In several series a substantial fraction of operations performed for suspected ischemic HPVG found no resectable transmural necrosis, so a surgical-case percentage cannot be read as a necrosis percentage (61). When this review refers to necrosis-driven HPVG, the referent is transmural infarction or histologically confirmed necrosis, not CT-suspected ischemia alone, not pneumatosis intestinalis, and not the mere fact of surgical exploration. These necrosis-driven presentations are typically acute events driven by primary vascular occlusion, non-occlusive mesenteric ischemia, or mechanical insult in often non-cirrhotic patients. In such settings, dysbiosis, whether pre-existing or rapidly induced by antibiotics, fasting, stress, or ileus, is proposed to act as a concurrent immune amplifier (hypothesis). Gas-forming organisms including *Clostridium perfringens*, *Escherichia coli*, and Klebsiella have been isolated in HPVG (direct clinical). Whether community-level dysbiosis is what permits their overgrowth has not been tested in HPVG cohorts. The original vascular or infectious insult remains dominant in necrotic disease. The I/R proxy data above show that even when the initiating event is vascular, the resident microbiota tunes the magnitude of barrier failure, neutrophil activation, and inflammation (15, 16). Concurrent pneumatosis intestinalis, when present, is an intramural finding that can raise the pre-test probability of transmural ischemia; it does not convert necrosis-driven HPVG into a different gaseous disease and it is not required for the diagnosis. When HPVG is detected in a patient who already has cirrhosis or PSVD, portal hypertension is recorded as a host covariate that may modify barrier geometry and post-entry washout. It is not treated as a third validated HPVG phenotype, it is not treated as an independent generator of intraportal gas, and dysbiosis in that host is not classified as an upstream driver of the radiographic sign. Raised downstream mesenteric or portal venous pressure would, other things equal, reduce rather than increase the lumen-to-vein driving gradient at entry, as specified in Section 4.4. What chronic portal hypertension can plausibly change is route geometry through splanchnic congestion, mucosal edema, and GVB failure, and the later visibility of an already entered bolus through slow, bidirectional, or hepatofugal flow. The restricted joint-scaling hypothesis in Section 4.4 is therefore not assigned to acute necrosis-driven HPVG and is not assigned to isolated iatrogenic or pressure-related HPVG; if tested at all, it belongs only to a necrosis-excluded, non-iatrogenic window in which portal hypertension, when present, remains a covariate rather than a phenotype label. Portal vein thrombosis in the same host is an occlusive vascular event, not a gaseous equivalent of HPVG.

Acute necrotic and critically ill HPVG presentations add three confounders that can each produce a low-diversity, Enterobacterales-enriched community without any chronic portal-hypertensive history, and that therefore prevent emergency-day stool or mucosa from being read as a pre-morbid setpoint (arrow C in Section 3.2). First, anti-anaerobic antibiotics deplete butyrogenic obligate anaerobes and favor pathobiont domination on a timescale of days; cumulative exposure to agents such as piperacillin–tazobactam, amoxicillin–clavulanate, metronidazole, or meropenem is associated with loss of butyrogenic taxa and expansion of Enterococcus, Streptococcus, or Escherichia–Shigella in intensive-care cohorts (63) (indirect clinical). A cultured, pathway-defined isolate of *Clostridium perfringens* or *Escherichia coli* remains direct clinical evidence for a bacterial route of entry. It does not prove that community-level dysbiosis caused that overgrowth when the sample was obtained after empiric therapy. Consensus washouts of about four weeks after antibiotics are incompatible with the emergency window, so any future HPVG protocol must timestamp the index CT against the antibiotic clock and must not treat an on-antibiotic emergency sample as the fermentative term of the Section 4.4 statement. Second, intestinal ischemia–reperfusion itself injures epithelium, raises luminal oxygen, and tunes neutrophil recruitment and NETosis; germ-free or antibiotic-depleted states can exaggerate lipopolysaccharide-triggered NET formation in that model (16) (preclinical). I-FABP tracks enterocyte injury and portal hemodynamics and is not a dysbiosis readout and is not a GVB readout (64). Enterobacterales expansion in necrotic HPVG is therefore first interpreted as an ischemic niche plus antimicrobial selection, not as proof of a pre-existing community collapse. Third, critical illness, nil-by-mouth status, opioids, acid suppression, vasopressors, and ileus collapse colonization resistance within hours, and experimental brain death rapidly reshapes both the intestinal microbiota and the hepatic metabolome (9) (indirect clinical and preclinical). Emergency-day sequencing in HPVG therefore records a concurrent state. After transmural necrosis has been excluded, and after isolated pressure-related or iatrogenic entry has been set aside, a later off-antibiotic recovery sample is required before compositional data can be tested as a research covariate of subsequent course.

### 3.4 Conflicting and negative immunological evidence

Inconsistencies must be acknowledged. A 2025 cross-sectional study found no significant association between gut-microbiome composition and portal vein thrombosis in end-stage liver disease (25), highlighting the influence of disease stage, sequencing methodology, and confounding comorbidities. That study answers an occlusive-thrombosis question. Portal vein thrombosis is not intraportal gas, is not pneumatosis intestinalis, and is not used here as a surrogate manifestation of HPVG. Such data underscore that dysbiosis is neither necessary nor sufficient for HPVG, but likely acts as a modifiable immune risk amplifier within a multifactorial causal web. Heterogeneity in sampling site (stool versus mucosa versus blood), sequencing depth, and case definition further complicates cross-study comparison and cautions against over-interpreting any single microbial or immunological signature (56, 57). Negative and positive findings are not necessarily in conflict once the amplifier-versus-driver stratification is applied. A null association in one endpoint, for example portal vein thrombosis in end-stage disease, where fixed vascular pathology dominates, is fully compatible with a contributory immune role in another endpoint, for example barrier failure, endotoxemia, and gas-forming overgrowth in subacute, non-necrotic HPVG. Interpreting the literature therefore requires matching each study’s population and endpoint to the specific immunological claim being tested, rather than seeking a single universal verdict on whether dysbiosis causes HPVG. A null stool-microbiome association with portal vein thrombosis in end-stage liver disease answers an occlusive question and cannot adjudicate arrows A–C in Section 3.2, and an intensive-care or post-antibiotic community in acute HPVG cannot be used as evidence that chronic, pre-morbid dysbiosis generated intraportal gas. Taken together, the proxy evidence base is biologically coherent and sufficient to justify the mechanistic dissection that follows. **Table 1** maps each principal claim by population, design, endpoint, and evidence category; later mechanistic sentences should be read against that map.

## 4 Innate immune and barrier pathways from dysbiosis to intraportal gas

This section traces a stepwise immunological chain from microbial imbalance to barrier failure, innate activation, and intraportal gas in phenotypes in which a microbial or innate contribution is biologically plausible (**Figure 3**). Individual links are supported in proxy systems; the assembled sequence ending in radiographic HPVG is hypothesized and is not required for iatrogenic or purely pressure-related entry. Every major arrow in **Figure 3** should be read with its evidence label.

**Figure 3:**
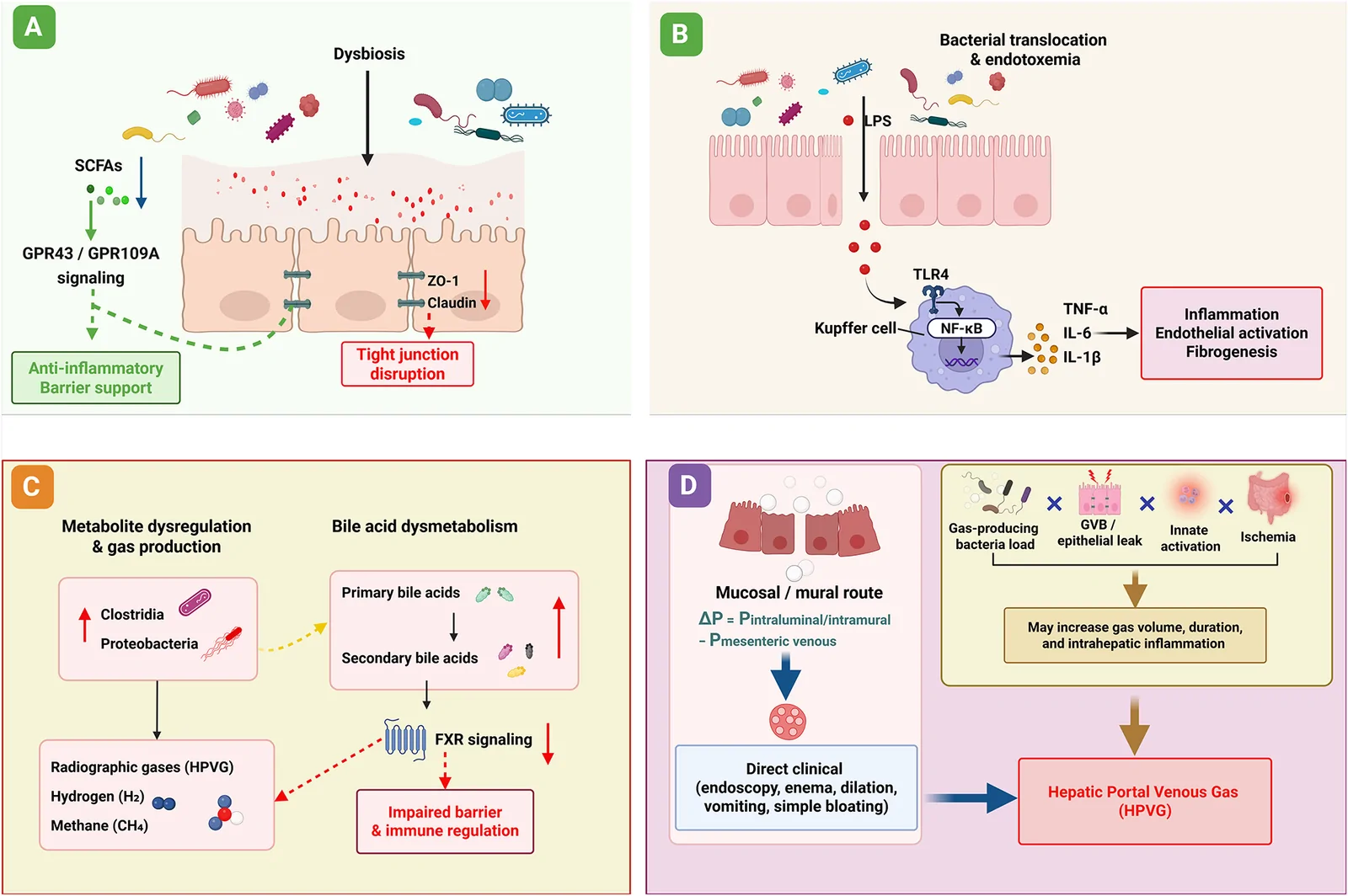
Proposed mechanisms linking gut dysbiosis to HPVG. **(A)** Dysbiosis reduces SCFA signaling and weakens epithelial barrier integrity. **(B)** Bacterial translocation and innate immune activation in the gut - liver axis. **(C)** Metabolic dysregulation and gas production by fermentative pathways. **(D)** Mucosal or mural entry plus a local driving gradient, with optional immune amplifiers leading to radiographic HPVG. Panels that summarize proxy-disease immunology are indirect clinical or preclinical; the assembled sequence ending in adult HPVG is hypothetical. Reduced SCFAs weaken GPR43/GPR109A signalling and downregulate ZO-1 and claudins (indirect clinical/preclinical). Commercial zonulin assays are not shown, because they do not measure authentic pre-haptoglobin-2 and are not used here as a marker of paracellular leakiness. Translocated LPS primes Kupffer-cell TLR4–NF-κB and cytokine release (indirect clinical in cirrhosis and related disease). Fibrogenesis is a chronic proxy readout, not required for gas entry; genotypically defined hvKp loci are omitted because they have not been shown in adult HPVG. Mixed-acid PFL–FHL fermentation, 2,3-butanediol fermentation, and clostridial PFOR–hydrogenase activity may yield fermentative H_2_ and CO_2_; methanogenesis may convert H_2_ plus CO_2_ to CH_4_. These pathways define a gas-forming isolate. They do not identify the CT lucency as H_2_/CO_2_, and they are not a depiction of emphysematous infection or of pneumatosis. FXR signalling modifies barrier tone rather than gas production. Iatrogenic N_2_/CO_2_ can cause HPVG without this microbial arm (direct clinical). A mucosal or mural route plus a local driving pressure gradient is sufficient after endoscopy, enema, dilation, vomiting or isolated distension (direct clinical). Fermentative load, GVB or epithelial leak, innate activation and ischemia are optional amplifiers of volume, duration and hepatic inflammation (hypothesis). They are not a required two-hit, and the joint-scaling hypothesis that radiographic gas burden may rise when fermentative output and GVB leak are both increased is confined to necrosis-excluded, non-iatrogenic HPVG. Portal hypertension is a post-entry modifier of washout or CT visibility, not a source of gas. Variable CT sensitivity and asynchronous sampling are measurement confounders of that hypothesis, not additional biological hits. https://BioRender.com/xm2iq8n.

### 4.1 Immune-mediated barrier disruption and increased permeability

The intestinal epithelial barrier is the first immunological line of defense against luminal contents entering the portal circulation. Dysbiosis compromises it through interrelated immune and metabolic mechanisms. Reduced SCFA availability downregulates ZO-1 and claudins via diminished GPR43/GPR109A signaling and relief of histone-deacetylase inhibition, while depriving colonocytes of their principal energy substrate and weakening antimicrobial-peptide and IgA support (42–45). Human cirrhosis studies report lower fecal butyrate and increased intestinal permeability as shown by elevated lactulose/mannitol ratios (11, 52). Circulating “zonulin” values in the same literature are almost always generated by commercial ELISAs that do not reliably quantify authentic zonulin/pre-haptoglobin-2 and instead capture complement-related or haptoglobin-related proteins, now often reported as zonulin-family peptides. Authentic zonulin is the uncleaved haptoglobin-2 precursor; commercial kits of the Immundiagnostik and CUSABIO class do not recover recombinant pre-haptoglobin-2, still generate signal in HP1/HP1 non-producers, and therefore cannot be read as a straightforward marker of paracellular leakiness (65–67). These permeability changes are graded rather than all-or-none, but the direction and slope are marker-specific rather than a universal monotonic rise across Child-Pugh or ACLF stages. Dual-sugar tests and selected circulating markers of epithelial injury can increase as barrier failure worsens, whereas zonulin-family peptides may fall in advanced cirrhosis because haptoglobin is hepatically synthesized (56). The PSVD series cited above for circulating LPS used the same class of commercial “zonulin” ELISA; those optical-density values are retained only as zonulin-family peptide immunoreactivity and are not used here as evidence of calibrated tight-junction failure. In a stage-stratified cirrhosis series, the GVB marker PV-1 and the translocation marker EndoCAb were higher in decompensated than in compensated disease without a further concentration increase in ACLF, I-FABP correlated with the portosystemic pressure gradient (Spearman r_s_ = 0.481) and fell after TIPS, and LBP did not follow a clean stage ladder (56). Those dissociations matter for interpretation: I-FABP reports enterocyte injury and tracks portal hemodynamics rather than GVB failure; LBP is an acute-phase LPS-binding protein and is not gut-specific; circulating PV-1 was measured with a research ELISA, was often near the assay floor in compensated disease, was not enriched in portal versus hepatic venous blood, and is not a standardized clinical assay. The magnitude of endotoxin delivery to the liver still appears to scale with the degree of barrier compromise in proxy studies, providing a plausible dose–response link between dysbiosis severity and downstream innate activation in those diseases (56, 68). Blood-based surrogates can therefore be used in cirrhosis research to interrogate complementary layers of barrier failure, with the GVB remaining a distinct immune compartment that can fail after the epithelial barrier (55, 56, 69). They do not constitute a single, interchangeable “permeability panel,” and none of these markers has been qualified in adult HPVG. Pathological bacterial translocation, first characterized in cirrhosis two decades ago, occurs early in disease and triggers selective immune activation even before decompensation (70, 71). In PSVD, chronic low-grade PAMP translocation sustains LSEC capillarization, including loss of fenestrations and basement-membrane deposition, and activates HSCs, thereby raising intrahepatic resistance (12, 13). Epithelial repair can also be impaired when FXR-agonistic bile-acid signaling in enterocytes is lost, because that pathway supports proliferative, anti-apoptotic, and antimicrobial programs (43, 47). This statement concerns receptor-dependent epithelial repair in proxy disease. It does not treat every secondary bile acid as protective (47, 51). Increased permeability has been conceptualized as a rheostat that scales endotoxin delivery to hepatic innate cells, and endotoxin translocation tracks with hard clinical events such as variceal bleeding (68, 72). Collectively, these alterations create a permissive immune environment for bacteria and PAMPs to enter the mesenteric and portal systems in cirrhosis and PSVD (indirect clinical). We propose that the same permissive state could lower the threshold at which luminal gas enters once a mucosal or GVB breach is present (hypothesis). Direct measurements of these barrier-immune markers in adult HPVG have not been reported.

### 4.2 Bacterial translocation, pattern recognition, and innate immune activation

Once the barrier is compromised, viable bacteria and microbial products, including LPS, flagellin, and peptidoglycan, translocate into the portal vein. In cirrhosis, bacterial translocation is detectable in a substantial proportion of patients and correlates with systemic endotoxemia (52, 53, 70). LPS–Toll-like receptor 4 (TLR4) engagement on Kupffer cells and LSECs triggers NF-κB activation and release of tumor necrosis factor-α (TNF-α) and interleukin-6 (IL-6), amplifying hepatic inflammation, neutrophil infiltration, oxidative stress, and endothelial dysfunction (52, 54). TLR4–NF-κB signaling and NLRP3 inflammasome activity are not treated here as a single linear cascade. In the canonical two-signal model, TLR4 ligation is primarily a priming or licensing event that upregulates NLRP3 and pro-IL-1β. Inflammasome assembly and caspase-1–dependent maturation of IL-1β and IL-18 typically require a distinct activation step, such as potassium efflux, mitochondrial reactive oxygen species, or other damage-associated signals generated by ischemia or necrosis. Non-canonical cytosolic LPS sensing via caspase-4/5/11 and an alternative TLR4–TRIF–caspase-8 route in human monocytes further separate these events by cell type. Where ischemia supplies that second signal, mature IL-1β and IL-18 can deepen tight-junction failure and recruit myeloid cells (52, 54). Recruited neutrophils contribute both phagocytosis and NETs. In acute mesenteric ischemia–reperfusion, the resident microbiota restrains NETosis while supporting neutrophil recruitment, so that germ-free or antibiotic-depleted states can exaggerate LPS-triggered NET formation. This is a context-dependent I/R observation and has not been demonstrated in patients with HPVG (preclinical) (16, 71). In PSVD, LPS–TLR4 signaling has been implicated in platelet activation, von Willebrand factor (vWF) release, and microvascular thrombosis, mechanisms that raise portal pressure in that disease (13, 14). Raised portal pressure is not hypothesized to facilitate *de novo* gas entry. The net result in proxy liver disease is a feed-forward innate loop in which inflammation further impairs barrier function and motility, perpetuating translocation (71). Quantitative correlations reported in proxy studies, for example between selected translocation or barrier markers and portal-pressure indices, or between dysbiosis severity and adverse clinical outcomes, support these links within portal hypertension and cirrhosis as associations rather than as calibrated effect sizes (indirect clinical; associative, not GRADE-rated). A coefficient of approximately 0.75 for LPS versus HVPG, and odds ratios of 2.5–3.2 for poor outcomes, could not be traced to a primary analysis with a defined population, endpoint, sample size, and confidence interval, and those numbers are therefore removed from the text and from **Figure 3**. Closest published coefficients describe different contrasts, including a Spearman correlation of 0.67 between the reduction in portal LPS and the reduction in portal-pressure gradient after TIPS in a small series, and the I-FABP–pressure correlation cited in Section 4.1; they are not interchangeable with a baseline LPS–HVPG law. These associations are proxy endpoints, not HPVG endpoints.

Reduced colonization resistance in dysbiosis can permit overgrowth of organisms that encode hydrogenogenic fermentation pathways (hypothesis as an upstream cause of that overgrowth in HPVG). Organisms isolated from blood or tissue in some radiographically confirmed HPVG cases include *Clostridium perfringens*, *Escherichia coli*, *Citrobacter*, *Candida*, and, less often, Klebsiella spp. (direct clinical). Consistent with the operational definition in the Introduction, “gas-forming” here means that pathway capacity, not gas detected in the clinical laboratory, not emphysematous infection, not pneumatosis, and not a chemical identification of portal gas. These isolates should be read by pathway, not by phylum. *Escherichia coli* generates H_2_ and CO_2_ by mixed-acid fermentation: pyruvate-formate lyase cleaves pyruvate to acetyl-CoA and formate, and the formate hydrogen lyase complex splits formate to H_2_ plus CO_2_. *Clostridium perfringens* lacks a complete tricarboxylic-acid cycle and respiratory chain; pyruvate–ferredoxin oxidoreductase releases CO_2_, ferredoxin-hydrogenase releases H_2_, and acetyl-CoA is dissimilated to acetate, ethanol, lactate, and butyrate. The histotoxic loci *plc*/*cpa* (α-toxin) and *pfoA* (perfringolysin O) can enlarge a mural route of entry but are not the enzymes that synthesize gas. *Klebsiella pneumoniae* and *K. oxytoca* ferment glucose to hydrogen and carbon dioxide through mixed-acid and 2,3-butanediol pathways; this metabolic capacity is a species-level property of many Enterobacterales and is not conferred by the hypervirulence plasmid. A small number of adult HPVG reports have recovered untyped *Klebsiella pneumoniae* or *K. oxytoca* from blood, pus, stool, or gastric fluid, typically in necrotic colorectal tumor, emphysematous gastritis, or immunosuppressed ileitis (direct clinical, case-level). Emphysematous gastritis in those case reports is an intramural or mural emphysematous infection (construct B) that may coexist with HPVG; it is not the definition of a gas-forming isolate. None of these HPVG isolates has been shown to carry the genotypic marker set that currently best defines hypervirulent *Klebsiella pneumoniae* (hvKp), namely *iucA*, *iroB*, plasmid-borne *rmpA* or *rmpA2*, and *peg-344*.

Hypervirulent *Klebsiella pneumoniae* is a well-characterized cause of community-acquired pyogenic liver abscess and metastatic infection after gut-to-portal translocation of viable organisms (indirect clinical). That pathway shares the portal vein as an anatomic conduit with HPVG; it has not been shown to generate free intraportal gas from injured bowel. Gas within a Klebsiella liver abscess, hepatic venous gas after abscess rupture, and septic portal-vein thrombophlebitis are not equivalent to bowel-derived HPVG. They are neighboring portal-axis infections and are not interchangeable with HPVG; this review does not treat abscess-cavity gas or hepatic venous gas as bowel-derived HPVG. We therefore treat ordinary pathway-defined Klebsiella as an uncommon cultured contributor to the bacterial route of HPVG, and we do not treat hvKp immune-evasion determinants as an HPVG mechanism (indirect clinical for pyogenic liver abscess; not demonstrated in HPVG). Virulence genes and fermentative gas genes are therefore separate axes: *plc* and *pfoA* may lower the threshold for intravascular entry; *iucA*, *iroB*, *rmpA*/*rmpA2*, and *peg-344* explain survival after translocation in liver-abscess biology; only PFL–FHL, PFOR–hydrogenase, and 2,3-butanediol fermentation explain microbial H_2_/CO_2_ production.

### 4.3 Metabolite dysregulation, fermentative gas, and immune–vascular remodeling

Beyond barrier and innate-sensing effects, dysbiosis alters the luminal metabolome in ways that both weaken immune defense and increase fermentative gas. Loss of acetyl-CoA butyrate-production capacity reduces butyrate-mediated anti-inflammatory and barrier-supportive signaling and diminishes colonization resistance against hydrogenogenic fermenters (45). The gas that ultimately appears in the portal vein may be a microbial product. That statement is a hypothesis about source, not a chemical identification of HPVG gas; the molecular composition of intraportal gas has not been measured, whereas hydrogen-rich gas has been sampled only from pneumatosis-cystoides cysts, an intramural compartment distinct from the portal vein. Bacterial fermentation of undigested carbohydrate and protein yields hydrogen and carbon dioxide, which cross-feeding methanogenic archaea (for example, *Methanobrevibacter smithii*), sulfate-reducing bacteria, and reductive acetogens convert to methane, hydrogen sulfide (H2S), or acetate, respectively. In a balanced ecosystem, hydrogen is efficiently consumed and net gas volume stays low. Dysbiosis that expands mixed-acid, 2,3-butanediol, or clostridial PFOR–hydrogenase capacity while depleting hydrogenotrophic methanogenesis, sulfate reduction, or acetogenesis shifts the community toward net gas accumulation (4). Overgrowth of these taxa elevates hydrogen, methane, and other VOCs, which can raise intraluminal pressure and favor migration into the bowel wall and portal system once immune barriers have failed. Pathway-defined fermentative isolates (C. perfringens) and facultative organisms (E. coli and, in isolated cases, untyped *Klebsiella pneumoniae* or *K. oxytoca*) are prolific gas producers, particularly where mucosal disruption or necrosis is present. Contemporary case series and reviews support an enabling role of such isolates once mucosa is injured (direct clinical for selected cultured taxa; hypothesis for community-level dysbiosis as the upstream cause of that overgrowth). Prolific gas production by Klebsiella reflects carbohydrate fermentation, not genotypically defined hypervirulence. That enabling role is sufficient in some infectious or necrotic cases; it is not necessary in iatrogenic or purely pressure-related HPVG, where the entering gas may be swallowed nitrogen or insufflated air or carbon dioxide rather than a product of pathobiont fermentation (direct clinical). Luminal fermentative gas, intramural gas, and portal venous gas are successive compartments: a gas-forming isolate may increase the volume available to cross a mural defect, but intramural pneumatosis is not required for the definition, and portal lucencies remain an imaging sign rather than an assayed microbial product. These compartments are therefore related by anatomy, not by identity: pneumatosis intestinalis is not HPVG, and neither finding is pyogenic liver abscess or portal vein thrombosis.

In the hepatic vascular compartment, microbial products and selected metabolites can amplify innate endothelial activation in cirrhosis and PSVD. The relevant upstream microbial change in those proxy diseases is facultative-anaerobe expansion supported by leaked oxygen and inflammation-derived nitrate or S-oxides, not a phylum-level Proteobacterial law. The best-supported mediator in those proxy diseases remains gut-derived LPS acting on TLR4 on LSECs, Kupffer cells, and HSCs, with loss of LSEC fenestrations, basement-membrane deposition, stellate-cell activation, reduced nitric oxide, and microvascular thrombosis via von Willebrand factor release. These immune–vascular changes elevate portal pressure in cirrhosis and PSVD (indirect clinical). Trimethylamine N-oxide is a gut-derived metabolite oxidized in the liver, but the mechanistic literature linking TMAO to endothelial injury and thrombosis comes chiefly from systemic cardiovascular models. Liver-specific reports are mixed and include both LSEC-injurious and LSEC-protective findings. TMAO is therefore not used here as a pathway to HPVG-specific portal vascular remodeling or intraportal gas (73, 74). Hydrogen sulfide is likewise not treated as a unidirectional toxin. Low-concentration, host-derived H2S can support barrier tone and serve as a mitochondrial substrate, whereas high-concentration, microbially generated H2S can inhibit respiration and injure epithelium; the sign depends on concentration, enzymatic source, and compartment. No H2S measurement links this dual biology to radiographic HPVG. Portal-hypertensive vascular remodeling is indirect clinical in cirrhosis and PSVD. Its proposed relevance to gas entry versus post-entry clearance is specified in Section 4.4 and is not treated here as an independent generator of HPVG.

### 4.4 A physical-core model of intraportal gas entry

A mechanistic account of HPVG must ultimately explain how gas physically crosses from the bowel lumen into portal venous blood, the step that distinguishes HPVG from asymptomatic intestinal gas. Classically, this step has been explained by a mechanical theory (mucosal injury and raised intraluminal pressure) and a bacterial theory (local production or tracking of gas by pathway-defined fermentative isolates), which are not mutually exclusive and do not have to operate together (direct clinical). Iatrogenic HPVG after upper endoscopy, balloon dilation, colonoscopy, contrast or saline enema, vomiting-induced gastric emphysema, acute gastric dilatation, ileus, and isolated bowel distension demonstrates that ordinary luminal or insufflated gas, which is largely nitrogen and carbon dioxide rather than a pathobiont-specific fermentative product, can enter through a pressure-driven mucosal defect without documented community-level dysbiosis, a dominant pathway-defined fermentative pathobiont, or a substantial PAMP load (direct clinical). Cadaver experiments in which applied intraluminal air pressure produced pneumatosis and portal gas across ulcerated mucosa further show that a contemporaneous microbial gas source is not required for physical entry. Those experiments also show why intramural gas and portal venous gas cannot define “gas-forming”: both can be produced by applied air in the absence of any isolate.

We therefore recast the working model as a physical core plus optional immune–microbial amplifiers (hypothesis), rather than as an immune two-hit requirement in which a barrier breach must coincide with a microbial gas source and PAMP load. The two-hit pairing of GVB or epithelial breach plus a microbial gas–PAMP source is therefore not the necessary core of this review; it is an optional amplifier layer that may increase volume, duration, and inflammatory cost in selected non-iatrogenic, non-necrotic presentations. The quantitative joint-scaling statement that follows is not a restatement of a required two-hit model. The necessary physical conditions are a route of entry and a local driving pressure gradient from the lumen or bowel wall into mesenteric veins. Elevated intraluminal pressure from insufflation, fermentation, ileus, obstruction, or vomiting, combined with mucosal ulceration, biopsy or dilation injury, ischemic epithelial injury, or proposed GVB failure, can allow gas to dissect into the submucosa and to track along mesenteric venules as pneumatosis, from which it drains centripetally toward the liver (4)(mechanical route: direct clinical; GVB as a candidate last checkpoint in selected HPVG phenotypes: hypothesis). Pneumatosis in this sequence is the intramural transit compartment, not a synonym for a gas-forming organism. It is also not a synonym for HPVG, portal hypertension, portal vein thrombosis, or pyogenic liver abscess. Cadaver pressure experiments and iatrogenic series show that intraportal gas can appear without intramural gas, so pneumatosis intestinalis is neither necessary nor sufficient for the radiographic diagnosis of HPVG.

Entry and clearance are opposite hemodynamic problems and are not combined here into a single “favorable pressure or flow gradient.” Gas entry is governed by a local driving gradient, approximately the difference between intraluminal or intramural pressure and mesenteric venous pressure, together with a route of entry. Entry is favored by a rise in intraluminal or intramural pressure relative to mesenteric venous pressure. Clinically significant portal hypertension raises downstream mesenteric and portal venous pressure and would, other things equal, reduce rather than increase that driving gradient; elevated portal pressure is therefore not hypothesized to facilitate mechanical entry of luminal gas unless luminal or intramural pressure rises further or the mural defect is large enough that pressure is no longer rate-limiting (hypothesis constrained by basic hydraulics; iatrogenic and cadaver pressure experiments remain direct clinical for pressure-driven entry when downstream pressure is not similarly elevated). What chronic portal hypertension can plausibly change at the entry step is the route, not the downstream pressure: splanchnic congestion, mucosal edema, GVB failure marked by PV-1 in cirrhosis, and impaired epithelial repair are candidate ways of enlarging or multiplying pathways through which gas, organisms, and PAMPs could pass once a second hit is present (indirect clinical in cirrhosis and PSVD; hypothesis when applied to HPVG). After gas has already entered the portal tree, reduced portal velocity, commonly below 16 cm/s in portal hypertension, and in advanced disease bidirectional, stagnant, or hepatofugal, could slow washout of a finite gas bolus and thereby prolong the interval during which intrahepatic gas remains above the CT detection threshold (Doppler description of slow portal flow: indirect clinical in portal hypertension; radiographic persistence of HPVG as a function of velocity or HVPG: hypothesis). The opposite hemodynamic prediction must be stated with equal weight: hepatofugal flow and portosystemic collaterals can divert gas away from intrahepatic peripheral portal radicles, which are the classic CT territory of HPVG, and gas within collaterals has been imaged in cirrhotic bleeding without mesenteric ischemia. No study has correlated HVPG or portal-vein velocity with semi-quantitative CT gas burden or with time-to-resolution of HPVG. Slow portal flow is therefore not cited as evidence that portal hypertension independently generates portal gas; it is cited only as a testable modifier of visibility after entry. Portal hypertension remains a hemodynamic host state. It is not an infectious manifestation, not a thrombotic equivalent of pylephlebitis, and not a radiographic synonym for HPVG or pneumatosis intestinalis.

Optional biological amplifiers may increase the volume, duration, and inflammatory cost of an entry event but are not hypothesized to be universally required. Mucosal necrosis, bacterial invasion of the vessel wall, and locally generated gas from vessel-adjacent organisms may lower the threshold for intravascular entry and may supply PAMPs that amplify hepatic innate injury (bacterial route: direct clinical for cultured gas-formers). Immune-evasion biology described for genotypically defined hvKp explains survival in the portal circulation and abscess formation; it has not been shown to cause bowel-derived intraportal gas and is not invoked here as an HPVG amplifier (indirect clinical for pyogenic liver abscess; hypothesis if applied to HPVG). Dysbiosis is proposed to contribute to these amplifiers when it is present: gas-forming overgrowth could supply volume, pressure, and innate ligands, while epithelial and GVB immune failure could enlarge or multiply the route of entry (hypothesis). In iatrogenic or purely pressure-related HPVG these amplifiers may be absent. In acute necrotic HPVG they are proposed to act chiefly as concurrent amplifiers of a dominant vascular or mechanical insult. In a patient who already has chronic portal hypertension they could act further upstream by promoting GVB failure and by slowing or redistributing portal flow, but only as modifiers of a conventional entry event (host context as defined in Section 3.3).

We further state a restricted quantitative relationship as a testable immunological hypothesis rather than as an established equation or as a required two-hit law. This statement applies only to non-iatrogenic HPVG after transmural necrosis and isolated pressure-related or iatrogenic entry have been excluded. It is not proposed for acute bowel-necrosis-driven HPVG, in which mural disruption and local gas generation are dominated by infarction. It is not proposed when the imaged gas is ordinary luminal or insufflated N_2_ or CO_2_. In that necrosis-excluded, non-iatrogenic window, portal-gas radiographic burden, defined here as a pre-specified semi-quantitative CT grade on a stated reconstruction and contrast phase, could be higher when two independently measurable factors are both increased: the net fermentative gas-production rate of the luminal community, indexed by breath hydrogen and methane output under a protocol that records recent antibiotics, fasting, and bowel preparation, or by the relative abundance of hydrogenogenic and hydrogenotrophic taxa, and the degree of GVB compromise, indexed by tissue PV-1 immunohistochemistry or, if circulating PV-1 is measured at all, a research ELISA with a reported limit of detection and paired portal–peripheral sampling. Commercial zonulin-family peptide assays and I-FABP are not used as the GVB term of this hypothesis: I-FABP reports enterocyte injury and is reserved for the necrosis-entry arm. Portal flow stasis secondary to an elevated HVPG could act as a third permissive factor that reduces clearance of any gas that has already entered the portal system; the same raised downstream pressure is not hypothesized to generate intraportal gas or to enlarge the lumen-to-vein driving gradient at entry. None of these indices has been correlated with CT gas burden in an HPVG cohort. A multiplicative “product” form is one untested parametric possibility among additive, threshold, and interaction alternatives; no coefficient has been fitted.

Boundary conditions and confounders that would bias any test of the statement are therefore stated explicitly. First, portal haemodynamics: velocity commonly below 16 cm/s in portal hypertension, together with biphasic, stagnant, or hepatofugal flow and portosystemic collaterals, can prolong, dilute, or divert an already entered bolus and thereby change CT visibility without changing fermentative output or GVB leak. Second, variable CT detection sensitivity: collimation, reconstruction kernel, contrast phase, left-lobe peripheral distribution used to separate HPVG from pneumobilia, inter-reader grade, and the sensitivity gap versus plain radiography and versus bedside ultrasound when examinations are separated in time mean that “gas burden” is an imaging visibility score, not a molar quantity of microbial H_2_ or CO_2_. Third, variable sampling timing in acute illness: breath tests, stool or mucosal sequencing, and barrier assays obtained hours to days after the index CT, often after empiric antibiotics, fasting, nasogastric decompression, or bowel preparation, do not represent the community or the GVB state at the moment gas became visible; consensus washouts of about four weeks after antibiotics are incompatible with emergency sampling, and breath hydrogen is not mesenteric or portal flux because hydrogenotrophic methanogenesis and sulfate reduction consume H_2_. Fourth, intramural pneumatosis is a transit compartment that can inflate or deflate apparent portal-gas load and is neither GVB leak nor HPVG.

This formulation generates three falsifiable predictions, each confined to the necrosis-excluded, non-iatrogenic window defined above. First, patients with high-fermentative, low-diversity microbiomes and elevated GVB biomarkers would be expected to exhibit higher semi-quantitative CT gas scores even in the absence of frank necrosis, provided the gas source is microbial rather than isolated insufflation. Second, restoration of SCFA-producing taxa or reduction of gas-forming pathobionts could decrease subsequent portal-gas events in non-necrotic, non-iatrogenic HPVG. Third, GVB-targeted immunomodulation would be predicted to be more relevant as an adjunct in a chronic portal-hypertensive host after necrosis has been excluded than in purely acute ischemic HPVG or in isolated post-procedural HPVG. This prediction tests a host-context effect; it does not presuppose that chronic portal-hypertensive HPVG is an established phenotype. These predictions distinguish the present model from purely descriptive accounts of gas-forming bacteria and provide a concrete experimental roadmap for imaging-linked immune-omics studies. These predictions are for future testing and do not describe HPVG whose entire gas load is insufflated air or carbon dioxide.

### 4.5 Reverse causality and self-reinforcing immune injury

Reverse causality operates concurrently. Liver-derived alterations in bile-acid composition deplete immunoregulatory taxa and enrich gas-producing and pro-inflammatory organisms, closing a self-perpetuating loop (26, 51). Experimental and clinical observations support these bidirectional gut–liver immune interactions (26, 28), which help explain why, once initiated, dysbiosis-associated portal-hypertensive injury tends to be self-reinforcing. The convergence of barrier-immune failure, endotoxemia, metabolite dysregulation, and innate vascular remodeling, each reinforcing the others, is well described in chronic liver disease (indirect clinical). We propose that, past a threshold, such a loop could sustain a leaky GVB and alter portal flow in a chronic portal-hypertensive host and thereby make a subsequent conventional entry event more readily detectable as HPVG (hypothesis). The loop is not proposed to generate intraportal gas in the absence of the physical core defined in Section 4.4.

## 5 Immune and barrier biomarkers for phenotype stratification

Dysbiosis severity predicts adverse immune and clinical outcomes across proxy conditions. In cirrhosis and portal hypertension, patients with the lowest microbial diversity exhibit substantially higher risk of HE or SBP, complications that are driven by translocation and innate activation (11, 53). Severe dysbiosis has been associated with worse composite outcomes in proxy cohorts, and age and concurrent immunosuppression amplify these effects. HPVG-specific prognostic immune data are lacking. Retrospective cohorts show that ischemia, infarction, or uncontrolled intra-abdominal infection predict conservative-management failure (direct clinical observation), but those series did not measure microbiome composition or barrier-immune biomarkers. Emergency-department series identify lactate and other ischemia laboratories as the dominant determinants of immediate outcome. No circulating barrier, translocation, or microbiome assay is a clinically validated HPVG triage test. The markers below are a construct-specific research menu, not a bedside panel.

Candidate research markers should be grouped by biological construct rather than pooled as a single barrier-immune panel (**Table 3**). Compositional and functional research markers include acetyl-CoA butyrate-pathway gene abundance (*but*, *buk*, *bcd*), facultative-anaerobe or nitrate-reductase gene load, hydrogenase or formate hydrogen lyase gene abundance, hydrogenotrophic indices (*mcrA*, *dsrAB*), reduced Shannon diversity, and expansion of Enterobacterales; none has been measured in adult HPVG. Enterocyte-injury markers, principally I-FABP, report villus epithelial damage and early ischemia. I-FABP is not a marker of community-level dysbiosis and is not a marker of GVB failure; in cirrhosis it correlated with the portosystemic pressure gradient and fell after TIPS without tracking PV-1 (56, 69, 75). In an HPVG work-up it belongs with lactate and base deficit as a potential necrosis-arm research adjunct, not with diversity scores. The only epithelial-permeability research construct retained here is the dual-sugar (lactulose/mannitol or equivalent) test. Commercial “zonulin” ELISAs do not reliably measure authentic zonulin/pre-haptoglobin-2 and have important specificity limitations; in decompensated cirrhosis the signal also falls with failing hepatic synthesis, so it cannot be used as a monotonic leak meter (56, 65, 66). Zonulin-family peptide kits are therefore listed in **Table 3** only as a historical surrogate used in some proxy papers, not as a paracellular-leak assay and not as an HPVG research endpoint. Translocation and innate-activation research markers, including LBP, soluble CD14, and EndoCAb, index microbial-product exposure and systemic innate activation. They are not gut-specific, rise in sterile inflammation and Gram-negative sepsis, and in advanced cirrhosis are confounded by hepatic synthesis, portal congestion, and the acute-phase response (56). GVB research markers must be separated by matrix. Tissue PV-1 immunohistochemistry remains the strongest construct for endothelial GVB diaphragms. Circulating PV-1 has been detected by research ELISA across cirrhosis stages and correlated with EndoCAb and LBP rather than with I-FABP, but the assay is not a standardized clinical test, many compensated samples sit near the limit of detection, and the intestinal origin of the circulating antigen is unproven (56). Metabolite research markers such as reduced SCFA levels index immunometabolic remodeling in proxy disease, not HPVG gas entry. Serum TMAO is not advanced as an HPVG research marker, because the supporting vascular evidence is remote from bowel-derived portal gas and is not liver-compartment specific (73). Clinical scores (Model for End-stage Liver Disease [MELD], Child-Pugh) and imaging (HVPG measurement, CT gas quantification) remain essential for acute triage. They do not capture an immune phenotype that distinguishes reversible amplifier biology from inevitable necrosis, but they, together with ischemia laboratories, are the only tools currently qualified for the first clinical decision. A composite of a barrier marker, a diversity metric, and a CT gas grade is not proposed as a near-term clinical index. Any future composite would first require assay standardization, HPVG-specific reference ranges, necrosis-stratified calibration against surgery and radiographic gas burden, and *a priori* exclusion of isolated iatrogenic cases (hypothesis). No such index is available for clinical use, and none of its components has been qualified in HPVG (hypothesis). Patients whose HPVG follows endoscopy, enema, balloon dilation, or isolated gastric dilatation, and who lack ischemia or sepsis, should be excluded from such research qualification *a priori*, because the physical core of gas entry is already explained and microbiota-directed testing is not indicated. Patients whose only “chronic” feature is portal hypertension should likewise not be assigned to a separate clinical index until HPVG frequency, etiology, and adjusted independence have been measured in cirrhosis and PSVD; portal hypertension may be recorded as a host covariate, not as a phenotype label (hypothesis). The same rule bars treating portal vein thrombosis, pneumatosis intestinalis, or pyogenic liver abscess as HPVG phenotype labels.

**Table 3:** Construct-specific status of candidate markers discussed in this review.

Marker	Biological construct	What the usual assay reports	Clinical status today	Status in adult HPVG	Permitted claim in this article
Lactate, base deficit	Systemic hypoperfusion and ischemia	Routine clinical chemistry	Standard emergency tests	Used in HPVG series as outcome correlates	Direct clinical adjunct for necrosis triage; not a microbiome marker
I-FABP	Enterocyte/villus injury	Research or selected clinical ELISA	Research and selected ischemia studies	Not measured	Research marker for the necrosis/entry arm; not dysbiosis or GVB
Dual-sugar permeability tests	Paracellular epithelial leak	Research protocol	Research only	Not measured	Mechanistic research only; not emergency-feasible
Commercial zonulin/zonulin-family peptide ELISA	Intended tight-junction regulator	Complement-related, haptoglobin-related, or other family peptides; not authentic pre-haptoglobin-2	Not a validated permeability test	Not measured	Historical or exploratory surrogate only; do not treat as pre-HP2
LBP, soluble CD14, EndoCAb	PAMP exposure and innate activation	Research ELISA; acute-phase and hepatic-synthesis confounded	Cirrhosis research	Not measured	Nonspecific innate-activation research markers
Circulating PV-1 ELISA	Intended GVB leak	Research kit values; often near the detection limit; source of antigen unproven	Cirrhosis research only; not an IVD	Not measured	Research surrogate; not a standardized clinical assay
Tissue PV-1 immunohistochemistry	Endothelial GVB diaphragm	Histology	Research	Not measured	Strongest GVB construct; not obtainable in routine acute care
Stool 16S or shotgun sequencing	Luminal community composition and, if shotgun or targeted gene assays are used, butyrate-pathway, hydrogenogenic, and hydrogenotrophic gene content	Research sequencing	Research	No adult HPVG cohort	Hypothesis. Phylum ratios and a Clostridia/Lactobacillus ratio are not permitted HPVG risk metrics. Pathway-gene abundance may be tested only as a research covariate after necrosis exclusion.
Circulating 16S “blood microbiome”	Mixed microbial DNA	Low-biomass 16S; kitome-vulnerable	Research; no core blood microbiome in large healthy cohorts	Not an HPVG mechanism	Do not treat as viable translocation
Breath hydrogen and methane	Luminal carbohydrate fermentation	Standardized for malabsorption/SIBO-IMO, not portal gas	Clinical in other indications	Not measured	Recovery-phase research adjunct only; not ED triage
Semi-quantitative CT portal-gas grade	Radiographic gas burden	Clinical imaging	Standard diagnosis of HPVG	The defining readout	Endpoint for future marker studies; not itself a dysbiosis biomarker

In the acute presentation, the first task is to exclude transmural necrosis. Lactate, I-FABP, base deficit, and imaging dominate that decision (69). The research readouts in **Table 3** have no role in assigning surgery, conservative care, or a microbiota-directed product. After necrosis has been excluded, and after isolated pressure-related mucosal injury has been set aside, these markers could be tested for incremental discrimination in a protocolized research setting. Timing and safety constraints on any later experimental intervention are given in Section 6.5.

A functional class complementary to immune biomarkers deserves mention: the fermentative output of the community. Because the defining lesion of HPVG is intravascular gas that may be luminal, microbial, or both, breath hydrogen and methane testing could index the optional microbial gas-volume amplifier in non-iatrogenic phenotypes (4) (hypothesis). Breath tests reflect intestinal fermentation; they have not been shown to measure mesenteric or portal gas flux, and they cannot distinguish insufflated air or carbon dioxide from fermentative hydrogen and methane. They are standardized for carbohydrate malabsorption and for suspected small-intestinal bacterial or methanogen overgrowth, not for intravascular gas; orocecal transit, hydrogen non-excretion, and hydrogen consumption by methanogens further limit any attempt to treat a single breath value as a quantitative portal-gas source term. Breath testing is therefore not an emergency triage tool. In acute illness, antibiotics, fasting, decompression, and bowel preparation further disconnect a later breath or stool result from the community present when HPVG was first imaged; any protocol that tests the Section 4.4 statement must therefore timestamp the index CT and the research assays and must not treat emergency-day sampling as a baseline fermentative term. Volatile-organic-compound profiling and targeted quantification of gas-forming taxa offer additional routes to a gas-production phenotype that no purely compositional marker captures. Semi-quantitative CT grading of portal-gas burden could be paired, in a planned mechanistic study, with construct-specific research readouts to test the prediction from Sections 4.3 and 4.4 that in necrosis-excluded, non-iatrogenic HPVG portal-gas load could scale with both fermentative capacity and GVB compromise. That pairing should use tissue or, if circulating PV-1 is measured at all, explicitly research-grade PV-1 together with an enterocyte-injury marker kept on the necrosis-entry arm, not a commercial zonulin or zonulin-family peptide ELISA as a surrogate for GVB leak, epithelial paracellular leak, or authentic pre-haptoglobin-2. These measures remain hypothetical for HPVG and would require prospective correlation with immune markers and outcomes. They are proposed as disease-specific research adjuncts, not as replacements for ischemia assessment or CT, and they are not proposed for triage of isolated post-endoscopic or post-enema HPVG or of acute necrosis-driven HPVG.

Assay limits for each construct are stated in **Table 3**. Until HPVG-specific qualification exists, these tests should not enter emergency algorithms that decide between operative and conservative care. The clinical events against which any future marker could be calibrated—mortality, therapeutic resection for histologically confirmed transmural necrosis, decomposed operative pathways, length of stay, and protocolized radiographic resolution of portal gas—are specified as primary and secondary endpoints in Section 7.2.1 and **Table 4**. Those events belong to observational natural-history and safety analyses. They do not convert a barrier, diversity, or fermentation assay into a bedside triage test.

**Table 4:** Primary and secondary endpoints for future HPVG-focused observational and interventional studies.

Domain	Study A: all-comer observational registry	Study B: necrosis-excluded interventional protocol
Purpose	Natural history, phenotype fractions, calibration of ischemia laboratories and CT against tissue necrosis, denominator for off-protocol product use	Safety and biological response after standard-of-care exclusion of transmural necrosis and of isolated iatrogenic or pressure-related entry
Primary clinical endpoints	Co-primary: (i) all-cause in-hospital mortality with 30-day and 90-day landmarks; (ii) therapeutic source control within 72 hours of the index CT, defined as resection of histologically confirmed transmural necrosis or infarction	Product-related safety through day 30 (day 90 sensitivity): new or worsening bowel ischemia or new resection after enrollment; product-related bacteremia or invasive infection; delivery-route injury; product-related serious adverse event leading to discontinuation
Secondary clinical endpoints	Emergency exploration within 24 hours; therapeutic resection versus non-therapeutic or negative laparotomy versus delayed rescue laparotomy versus palliation without operation; hospital and intensive-care length of stay with death as a competing risk; new organ failure at 24–72 hours; 30-day bacteremia or intra-abdominal infection; 30-day unplanned readmission	30-day and 90-day all-cause mortality; delayed rescue laparotomy after enrollment; hospital and intensive-care length of stay among survivors; 30-day infection and unplanned readmission
Imaging endpoints	Time to complete resolution of intrahepatic portal gas; change in a pre-specified semi-quantitative computed-tomography grade on a stated reconstruction and contrast phase. Imaging secondaries, not survival surrogates	Same measures as exploratory imaging endpoints only
Biological or research endpoints	Tertiary covariates only: pathway-gene abundance, recovery-window breath hydrogen and methane, tissue or research-grade plasmalemma vesicle-associated protein 1, dual-sugar permeability, intestinal fatty acid-binding protein on the necrosis arm, nonspecific pathogen-associated molecular pattern readouts	Secondary biological: early detection of administered taxa; durable engraftment only if a persistence window is pre-specified; change in one construct-specific research marker
Explicitly not permitted as a primary efficacy claim	A composite of barrier marker plus diversity plus computed-tomography gas grade used as emergency triage	Avoidance of laparotomy; powered mortality reduction; importation of hepatic-encephalopathy or hepatic-venous-pressure-gradient effect sizes

## 6 Microbiota-directed strategies as experimental research questions, not current HPVG therapy

No randomized or systematically enrolled observational study has tested probiotics, synbiotics, rifaximin, dietary fiber, FMT, bacteriophage, or next-generation probiotics against radiographic HPVG resolution, need for laparotomy, or survival. Proxy trials in cirrhosis and HE define what a future HPVG protocol would need to measure; they do not establish benefit in HPVG. Section 6 is a research agenda, not a treatment algorithm (**Table 5**).

**Table 5:** Comparison of microbiota-directed interventions studied in proxy gut–liver diseases and considered here only as experimental questions for HPVG.

Modality	Proposed mechanism	Highest available source	Key limitations
Probiotics/synbiotics	Compete with pathobionts; enhance SCFA production; restore tight-junction integrity; reduce LPS translocation and lower HVPG (76, 77, 79).	Indirect clinical: randomized trials and meta-analyses in cirrhosis and hepatic encephalopathy, not in HPVG. The HVPG pairwise estimate in one 2024 meta-analysis used two trials (66 participants).	High strain/dose variability (10^8^–10^10^ CFU); small per-arm samples; short follow-up; rare infection risk in immunocompromised.
FMT	Restore a healthy community; reduce bacterial translocation and TNF-α; improve MELD and diversity; reduce HE recurrence (80–83, 85).	Indirect clinical: randomized trials in cirrhosis with hepatic encephalopathy, including THEMATIC; no HPVG endpoint.	Donor-screening risk; delivery heterogeneity (capsule vs. endoscopic vs. enema); ethical/consent issues; limited long-term data.
Diet/drug modulation (high-fibre diet, rifaximin)	Increase SCFA; promote beneficial taxa; reduce gut-derived inflammation and HE episodes; lower HVPG in some cohorts (86–88).	Indirect clinical: dietary and rifaximin trials in cirrhosis and encephalopathy, not in HPVG.	Sustained free-living fibre change is often limited after protocolized intake ends; fibre-type heterogeneity; potential resistance; mostly short-term studies; no HPVG endpoint.
Emerging therapies (phages, next-generation probiotics, enzyme/lipid inhibitors)	Precisely target a cultured gas-former if identified; deliver defined barrier/SCFA functions; modulate lipid metabolism (89, 90, 94, 102).	Preclinical in liver-disease models; hypothesis for HPVG. No phase III HPVG trial. Phage data against hvKp or alcohol-producing *Klebsiella pneumoniae* address liver injury, not intraportal gas.	Resistance; narrow host range; rapid clearance; lack of phase III RCTs; safety and ethical uncertainty in humans; preclinical liver-disease concepts only; no HPVG isolate has been treated with phage.

No row is an HPVG indication. Highest available source is always a proxy disease. Phage and next-generation probiotics are preclinical. FMT safety data from THEMATIC apply to stable outpatients with prior overt hepatic encephalopathy, not to critically ill or immunocompromised patients with HPVG.

### 6.1 Probiotics and synbiotics as mucosal immunomodulators

Meta-analyses of randomized controlled trials report that probiotic and synbiotic mixtures—particularly high-potency multi-strain formulations containing Lactobacillus, Bifidobacterium and Streptococcus species—reduce HVPG and lower HE recurrence risk, with synbiotics appearing beneficial for metabolic and inflammatory endpoints (76, 77). The majority of the supporting trials employed the original high-concentration product that contained eight specific strains (*Lactobacillus paracasei* DSM 24733, *Lactobacillus plantarum* DSM 24730, *Lactobacillus acidophilus* DSM 24735, *Lactobacillus delbrueckii subsp*. *bulgaricus* DSM 24734, *Bifidobacterium longum* DSM 24736, *Bifidobacterium infantis* DSM 24737, *Bifidobacterium breve* DSM 24732 and *Streptococcus thermophilus* DSM 24731) at approximately 450 billion CFU per dose. It should be noted that post 2016 VSL#3 probiotic formulation differs from the De Simone Formulation, which was commercially available under the trademark VSL#3^®^ only until 2016 (78). An early landmark trial showed that synbiotic modulation of the gut flora improved minimal HE by increasing non-urease-producing Lactobacillus species and reducing endotoxemia (79). Contemporary meta-analyses in minimal HE and cirrhosis confirm consistent, if modest, benefit on HE-related endpoints (77). The mechanistic rationale is immunological and is coherent with the barrier framework of Section 4. Exogenous Lactobacillus and Bifidobacterium strains compete with pathobionts for mucosal niches, supply or induce SCFA, enhance regulatory immune tone, and reduce luminal ammonia and endotoxin, thereby acting on the same translocation and innate-sensing steps implicated in HPVG. Whether these effects translate into fewer portal-gas events is untested, and the modest, heterogeneous effect sizes counsel against overstating benefit. Randomized evidence cited here concerns hepatic venous pressure gradient and HE in cirrhosis (indirect clinical), not HPVG. Limitations include strain- and dose-dependent variability (commonly 10^8^–10^10^ colony-forming units), treatment durations of about 4–24 weeks, small per-arm samples, and a theoretical infection risk in profoundly immunocompromised patients. Heterogeneity is endpoint-specific rather than a single field-wide I²: in cirrhosis probiotic meta-analyses, I² is frequently very high for blood ammonia and circulating endotoxin (often >85%), whereas some overt-hepatic-encephalopathy incidence analyses report low I². The randomized HVPG signal is particularly thin (one 2024 meta-analysis pooled two trials and 66 participants). Recent syntheses stress strain-specific standardization and longer follow-up.None of these trials used HPVG resolution or need for laparotomy as an endpoint. Probiotics and synbiotics remain candidate tools only for a future non-necrotic, recovery-phase research protocol.

Those trial durations describe planned exposure in proxy studies. They do not measure the time required for probiotic colonization, and they do not rank probiotics as kinetically faster than FMT. Most administered Lactobacillus and Bifidobacterium strains do not form a stably replicating mucosal population in adults. Fecal recovery during dosing is common, mucosal persistence is person- and strain-specific, and administered strains are often no longer detected within days after the last dose. Functional effects during gastrointestinal transit, including competition, metabolite supply, and change in innate tone, can therefore occur without durable colonization. Rare longer persistence has been documented for selected strains when an open niche is present; that exception is not a general property of the multi-strain products used in cirrhosis trials and is not an HPVG finding. Colonization is accordingly not treated here as a prerequisite for any proxy probiotic signal, and it is not used to time a future HPVG protocol.

### 6.2 FMT and restoration of hepatic immune tolerance

FMT is the most direct method for restoring a community capable of supporting mucosal and hepatic immune tolerance. Preclinical models show reduced bacterial translocation and TNF-α, and clinical work in cirrhosis reports safety with improved MELD and reduced HE recurrence (80–82). The evidence base has matured from a randomized trial showing that FMT from a rational stool donor improved HE and restored diversity (81), through a phase 1 randomized trial establishing the safety of oral FMT capsules in HE (82), to the pivotal THEMATIC phase II, double-blind, placebo-controlled trial (83).

THEMATIC enrolled patients with cirrhosis and prior overt HE already receiving lactulose and rifaximin. FMT, delivered as oral capsules with or without rectal enema from rationally selected donors, was safe and well tolerated, and donor engraftment tracked with baseline Lachnospiraceae abundance (83). Several microbiological and clinical endpoints were pre-specified as secondary or analyzed *post hoc*. In a *post hoc* analysis, overt HE recurrence was 40% with placebo versus 9% with any FMT (odds ratio 0.15; between-group p = 0.035). This secondary signal is best read as hypothesis-strengthening pending confirmation in an adequately powered trial (83). Together these trials provide the strongest randomized evidence to date for microbiome restoration in gut–liver-axis immune complications of cirrhosis, specifically HE (indirect clinical). The THEMATIC reduction in overt HE recurrence (40% with placebo versus 9% with any FMT; odds ratio 0.15) was a *post hoc* analysis in 60 outpatients and is not an HPVG effect size. It must not be used as an assumed effect size for radiographic portal-gas clearance or for avoidance of laparotomy. These data do not support clinical extension to HPVG. They may inform donor screening, route, and safety monitoring in a future protocol whose first endpoints would be safety and a pre-specified, time-stamped readout of donor-taxon detection or engraftment, kept distinct from radiographic gas clearance, rather than avoidance of laparotomy.

FMT is not biologically inert in the population that develops HPVG. Donor-derived extended-spectrum beta-lactamase *Escherichia coli* bacteremia after capsule FMT has been documented, including in a patient with cirrhosis treated for refractory HE, and one recipient died; that event prompted tighter donor screening for multidrug-resistant organisms (84). Additional hazards relevant to HPVG are procedural (aspiration with upper-gastrointestinal delivery; perforation or barotrauma with endoscopic or enema delivery across compromised bowel) and host-related (translocation across an already failed epithelial or GVB; concurrent broad-spectrum antibiotics that both select pathobionts and collapse colonization resistance). These risks can be discussed inside a regulated research protocol that uses screened, banked product and pre-specified stopping rules for bacteremia, aspiration, and new mucosal ischemia. They do not justify off-label adjunctive FMT in the emergency department or intensive care unit.

The HE-trial signal tracks with donor engraftment and recovery of SCFA-producing taxa more than with transient compositional change, implying that durable restoration of immunoregulatory function, not mere colonization, is what would matter if an HPVG trial were ever justified. That contrast is ecological, not a claim that FMT is intrinsically slower than probiotics. Stool community composition can shift toward the donor within days after FMT, particularly when antibiotics or an open niche reduce colonization resistance. Durable donor-strain engraftment and recovery of acetyl-CoA butyrate-pathway taxa remain variable and indication-dependent, and in THEMATIC they were assessed over weeks in stable outpatients already receiving lactulose and rifaximin. Many administered probiotic strains, by comparison, never colonize. Fecal shedding, early compositional shift, mucosal persistence, and durable functional restoration are therefore recorded as separate processes and are not collapsed into a single clock on which probiotics require days and FMT requires longer. Limitations include stringent donor screening, delivery-method heterogeneity, and limited long-term safety data beyond 6 months. A 2025 Chinese expert consensus conditionally endorses FMT for chronic liver disease provided rigorous donor screening and protocol standardization are observed (85). That consensus does not create an HPVG indication. Delivery route is itself a non-trivial immunological variable, because oral capsules and endoscopic or enema delivery may achieve different mucosal engraftment kinetics (81, 82). Route may change the probability and anatomy of early donor-taxon detection; it does not establish a universal latency that is longer than probiotic transit. For any prospective HPVG application, route, dose, and donor selection would need to be pre-specified rather than inherited unexamined from HE trials, given the distinct and, in acute cases, time-critical physiology at play.

### 6.3 Dietary fiber and gut-restricted antibiotics as complementary immune modulators

High-fiber diets increase SCFA production, promote SCFA-producing taxa, and reduce inflammatory markers. A randomized trial of high-fiber bread, for example, increased fecal SCFA-producing bacteria and microbial diversity under short-term, protocolized intake, with high adherence to the assigned bread during the two-week intervention periods (86, 87). That trial does not report a long-term compliance rate below 60%, and no such percentage is used here. Sustained high-fiber change in free-living adults is nevertheless often limited once study procedures end, which remains a practical barrier to dietary immunomodulation (indirect clinical; not an HPVG endpoint). Rifaximin, a minimally absorbed antibiotic, modestly increases microbial diversity, reduces HE episodes, and lowers HVPG in some cohorts. The mechanistic RIFSYS randomized trial demonstrated that rifaximin-α reduces gut-derived inflammation and endotoxemia in cirrhosis, supporting a microbiome-anchored immunomodulatory mode of action rather than simple bacterial suppression (53, 88). Both are low-cost adjuncts that require personalization based on baseline microbiome typing. Their contrasting immune mechanisms are instructive. Fiber works additively, feeding SCFA-producing taxa to rebuild barrier-supportive and regulatory functions from within. Rifaximin works subtractively yet selectively, suppressing pathobiont-driven innate inflammation without the wholesale diversity collapse caused by systemic antibiotics (53, 87, 88). If a future non-necrotic HPVG research protocol were to test both, they would be complementary rather than competing: rifaximin as a tool to reduce gut-derived innate inflammation, and fiber or other prebiotics as a tool to support barrier-immune recovery. That pairing is a testable design choice in proxy disease.

### 6.4 Emerging precision approaches to pathogenic immune ligands

Phage therapy targeting a dominant cultured gas-former, if one is identified in a given patient (for example C. perfringens or E. coli), offers conceptual precision unattainable with broad-spectrum antibiotics. Bacteriophages targeting cytolysin-positive *Enterococcus faecalis* attenuated alcoholic liver disease in mice (89), and phages against high-alcohol-producing *Klebsiella pneumoniae* alleviated steatohepatitis (90), establishing proof of concept for editing a pathogenic fraction of the microbiome in experimental liver disease while sparing commensals (91, 92). Those liver-disease models are not HPVG models, and no phage has been tested against an HPVG isolate. In confirmed infection-associated HPVG, narrow-spectrum targeting of a dominant cultured gas producer may complement restoration of barrier immunity, although resistance, narrow host range, and rapid clearance remain obstacles (92). hvKp-directed phage is relevant to pyogenic liver abscess research, not to current HPVG care or trial design (preclinical for liver disease; hypothesis for HPVG). Next-generation probiotics, including defined-strain biotherapeutics such as *Akkermansia muciniphila* and *Faecalibacterium prausnitzii*, are being developed to deliver specific barrier-fortifying and SCFA-dependent immunoregulatory functions with greater consistency than conventional formulations (93). Artificial-intelligence-driven multi-omics integration of metagenomics, metabolomics, and transcriptomics is beginning to yield reproducible microbiome-based classifiers (94). A universal gut-microbiome-derived signature predicts cirrhosis across cohorts (95), machine-learning models forecast fibrosis and cirrhosis from microbiome data (96), and broader reviews map the maturing role of these methods in liver-disease omics and microbiota analysis (97, 98). Such models may eventually predict immunological responder status and guide FMT dosing, although current performance remains preliminary. Phage targeting of a cultured gas-former and defined-strain next-generation probiotics remain preclinical concepts. The supporting experiments address liver injury in animals, not bowel-derived intraportal gas, and no phage has been tested against an HPVG isolate. They belong in the defined-consortium experiments outlined in Section 7.2, not in a bedside algorithm. In combination, multi-omic classifiers and barrier readouts might one day help researchers describe biological responder status inside a protocol that has already excluded necrosis by standard clinical means. They cannot assign individual HPVG patients to surgery, conservative care, or microbiota-directed treatment, and they must not replace imaging and ischemia assessment (hypothesis). No modality in **Table 5** has direct clinical evidence for an HPVG endpoint.

### 6.5 Timing and safety of immunomodulation in the acute abdomen

Preclinical acute mesenteric I/R models suggest that certain probiotics and commensals may reduce translocation and inflammation (59), yet human evidence is limited. In the PROPATRIA randomized trial, enteral multispecies probiotics in predicted severe acute pancreatitis did not reduce infectious complications and were associated with increased mortality (16% versus 6%; relative risk 2.53, 95% confidence interval 1.22–5.25) and bowel ischemia (9 versus 0 events, 8 fatal) (99). A secondary analysis of the same cohort found higher early enterocyte injury, measured as urinary I-FABP, in the probiotic arm, most evidently in patients with organ failure (100). This remains a decisive caution against broad, unselected microbiome intervention in acute abdominal emergencies. The mechanism is thought to involve increased local oxygen and metabolic demand in an already hypoperfused bowel, a scenario with clear parallels to necrosis-driven HPVG. Microbiota-directed experimentation should therefore never delay or replace operative management when bowel necrosis is present. It should not be introduced as a first-generation interventional trial whose efficacy target is avoidance of laparotomy. Any future test belongs in a non-necrotic, recovery-phase research protocol, and only after a prospective observational safety register has recorded off-protocol use, necrosis status, cultures, and 30-day ischemic and infectious events (85, 101). The endpoint hierarchy for that register and for any later interventional protocol is given in Section 7.2.1 and **Table 4**. In the observational register, all-cause in-hospital mortality and therapeutic source control are clinical co-primaries. In a first-generation interventional protocol the primary endpoint remains product-related safety, including new ischemia after enrollment. Surgical-exploration rate, hospital stay, and radiographic gas clearance are recorded, but they are not efficacy targets used to justify live-microbe administration in the acute abdomen.

### 6.6 Antibiotics: control of pathogenic innate ligands versus collateral dysbiosis

Antibiotics occupy a paradoxical position in this immunological framework. Empiric broad-spectrum coverage is indispensable in HPVG associated with sepsis, gas-forming infection, or suspected necrosis, and appropriately targets the translocating and gas-producing organisms discussed above. Yet the same agents deplete SCFA-producing commensals and collapse colonization resistance, potentially worsening the dysbiosis that predisposes to translocation and innate activation. This tension is especially consequential in critically ill patients receiving prolonged courses (10, 53). Two lessons follow. First, spectrum and duration should be minimized once cultures allow de-escalation, and narrow-spectrum or pathogen-directed strategies, including, prospectively, phage therapy against dominant gas producers, are conceptually preferable to sustained broad coverage that entrenches dysbiosis (92). Second, minimally absorbed gut-directed agents illustrate a more favorable immune profile: rifaximin reduces gut-derived inflammation and endotoxemia while modestly increasing diversity, functioning more as a microbiome modulator than as a decontaminant (53, 88). The practical implication for HPVG research is to pair necessary antimicrobial control of acute infection with a later, protocolized plan to study barrier-immune recovery, rather than treating antibiotics and microbiota-directed products as alternative treatments in the acute abdomen.

## 7 Discussion

### 7.1 An acuity-stratified immune framework for HPVG

HPVG remains first a radiographic sign whose established immediate mechanisms are mucosal disruption with raised luminal pressure and/or gas-forming infection, which can operate independently (direct clinical). In a subset of non-iatrogenic presentations it can additionally be viewed as the radiographic endpoint of the physical-core plus optional-amplifier model in Section 4.4 (**Figure 4**). The lower strip of **Figure 4** lists research questions only and is not a treatment algorithm. Host context follows Section 3.3: iatrogenic pressure-related entry does not require dysbiosis; necrosis-driven HPVG is dominated by the vascular or infectious insult; and portal hypertension, when present, is a modifier of barrier leak and clearance rather than a validated third phenotype.

**Figure 4:**
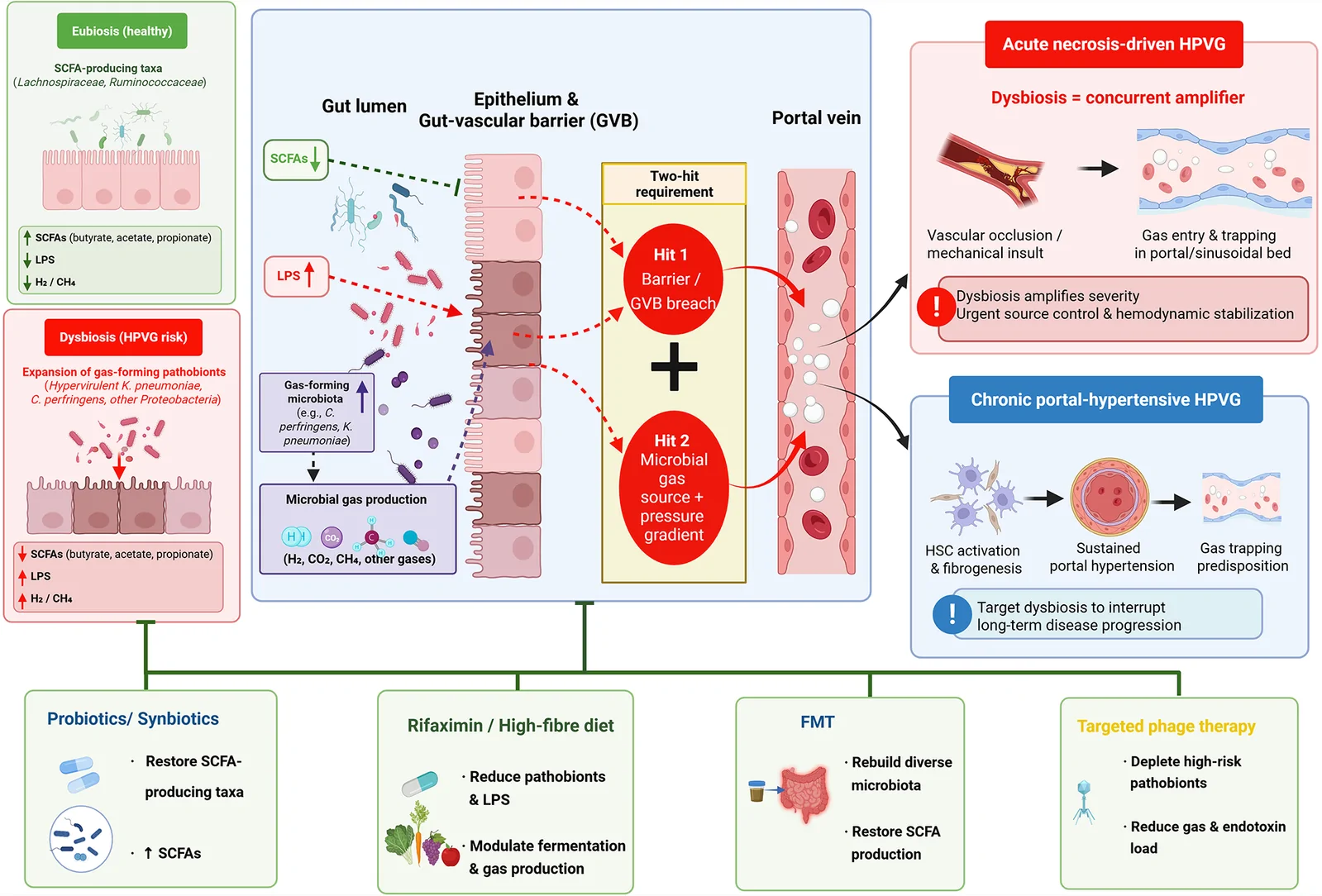
Optional microbial–immune amplifiers in hepatic portal venous gas, with research questions only. Left and centre: loss of SCFA support, LPS exposure, barrier or GVB leak, and pathway-defined fermentative isolates are shown as optional modifiers of portal-gas volume and inflammatory cost, not as co-requisites. Ordinary luminal or insufflated gas can enter after endoscopy, enema, dilation, vomiting, or isolated distension without dysbiosis (direct clinical). If the artwork still uses the label “gas-forming organisms,” that label means isolate-level fermentation capacity and does not mean emphysematous infection, pneumatosis, or assayed portal-gas chemistry. Genotypically defined hypervirulent *Klebsiella pneumoniae*, if still shown on the artwork, is a related gut–liver pathobiont and has not been isolated from radiographically confirmed adult HPVG. Right: acute necrosis-driven HPVG, in which dysbiosis is a proposed concurrent immune amplifier rather than the initiating vascular or infectious insult; and HPVG occurring in a host who already has portal hypertension, recorded as a covariate of route geometry and post-entry washout, not as a validated third phenotype and not as an upstream dysbiosis-driven generator of intraportal gas. Bottom: probiotics, rifaximin or diet, FMT, and phage are research questions with no HPVG outcome evidence. Emergency-day community profiles are not depicted as pre-morbid drivers, because antibiotics, ischemic mucosal injury, and critical illness can independently reshape the gut community in acute presentations. This figure is not a treatment algorithm. Created in BioRender. Bai, X. (2026) https://BioRender.com/g8jlbxf.

Candidate interventions remain research questions aligned with proxy-trial endpoints, not with HPVG triage. Proxy-trial numbers, including reduced HVPG, lower HE recurrence, and the *post hoc* THEMATIC contrast of 40% versus 9% (odds ratio 0.15), are cirrhosis results. They are not HPVG effect sizes and are not inputs to a clinical decision tree. Immunological assays do not modify the decision to operate. After necrosis and instability have been excluded, barrier and diversity markers may be recorded as research covariates of subsequent course.

### 7.2 From synthesis to experiment: six interlocking validation axes

Closing the principal evidence gaps will require coordinated progress along six interlocking axes, each linked to a falsifiable prediction of the model in Section 4.4.

1. Prospective, phenotype-stratified cohorts. Multicenter studies should enroll patients with HPVG or high-risk portal-hypertensive states (cirrhosis, PSVD), with standardized longitudinal microbiome and immune sampling at baseline and on days 7, 30, and 90, serial imaging, and HVPG measurement where feasible. Enrollment logs must record the antibiotic clock, anti-anaerobic spectrum, fasting or parenteral nutrition, nasogastric decompression, bowel preparation, vasopressors, acid suppression, lactate, I-FABP when obtained, and the interval between the index CT and each biosample. Comparator arms outside the acute HPVG window should follow the matrix in Section 3.2: cirrhosis with clinically significant portal hypertension; non-cirrhotic extrahepatic obstruction or PSVD with portal hypertension; PSVD or early disease without clinically significant portal hypertension; and non-diseased controls. Where a portosystemic shunt or other pressure-reducing intervention is already indicated for standard care, paired pre- and post-procedure samples may test arrow B but are not an HPVG treatment experiment. Outcomes must be stratified by the presence or absence of bowel necrosis, with sensitivity analyses addressing sequencing-platform and sampling-site heterogeneity (56, 57). Emergency-day samples are analyzed only as concurrent-state descriptions. Clinical co-primary endpoints for this all-comer observational registry are (i) all-cause in-hospital mortality, with pre-specified 30-day and 90-day landmarks, and (ii) therapeutic source control within 72 hours of the index CT, defined as resection of histologically confirmed transmural necrosis or infarction rather than any laparotomy. Key secondary clinical endpoints are emergency exploration within 24 hours; operative decomposition into therapeutic resection, non-therapeutic or negative laparotomy, delayed rescue laparotomy after an initial conservative pathway, and palliation without operation; hospital and ICU length of stay analysed with death as a competing risk; new or progressive organ failure at 24–72 hours; 30-day culture-proven bacteremia or intra-abdominal infection; and 30-day unplanned readmission. Time to complete radiographic resolution of intrahepatic portal gas, and change in a pre-specified semi-quantitative CT grade on a stated reconstruction and contrast phase, are imaging secondaries and are not treated as surrogates for survival. Prediction to be tested: in necrosis-excluded, non-iatrogenic HPVG, a recovery-window high-fermentative, low-diversity signature plus elevated GVB and innate-activation markers will independently predict higher pre-specified CT gas grade, delayed radiographic resolution, or a secondary clinical composite of death, new source-control failure, or unplanned readmission, after adjustment for portal velocity or HVPG, CT acquisition parameters, antibiotic exposure, and the interval between imaging and biosampling. This prediction is not tested in acute necrosis-driven HPVG and does not treat portal hypertension as a third HPVG phenotype.
2. Causal testing in controlled models. Gnotobiotic and defined-consortium colonization experiments, including selective introduction of gas-forming organisms that have actually been cultured in HPVG, principally C. perfringens and E. coli, and, as a separate arm, untyped *Klebsiella pneumoniae*, and reciprocal FMT, are needed to move beyond association. Consortia should be defined by pathway genotype: PFL–FHL-positive mixed-acid fermenters, PFOR–hydrogenase-positive C. perfringens, 2,3-butanediol fermenters, and a hydrogenotroph-replete comparator. A genotypically defined hvKp arm may be included only to test the negative control prediction that abscess-forming hypervirulence does not by itself produce bowel-derived intraportal gas. These models should quantify HPVG-relevant endpoints, including portal-gas volume by micro-CT or high-frequency ultrasound, portal pressure, barrier-immune markers, and innate cell activation, building on established intestinal I/R paradigms (15, 16). They should also include a purely mechanical arm in which defined luminal gas and raised intraluminal pressure are applied across an isolated mucosal defect in the absence of a fermentative consortium. Prediction to be tested: colonization with pathway-defined high-gas producers plus experimental GVB disruption will increase measurable intraportal gas and hepatic innate activation relative to a mechanical defect plus inert gas alone; a mechanical defect plus raised pressure is predicted to be sufficient for some intraportal gas even without high-gas producers. The original prediction that either hit alone will not produce gas is withdrawn, because it is incompatible with iatrogenic and cadaver pressure experiments (direct clinical). A further mechanical arm should test whether raising downstream mesenteric venous pressure, holding luminal pressure fixed, decreases rather than increases intraportal gas, which is the hydraulic prediction for portal hypertension at the entry step (hypothesis).

Acuity-stratified interventional research, only after an observational safety step. A first-generation interventional trial of FMT or phage should not use avoidance of laparotomy as an efficacy endpoint. A prospective multicenter register should first record whether any microbiota-directed product is given off protocol, together with necrosis status, cultures, and 30-day infective and ischemic events. Only after that safety denominator exists should an interventional protocol be considered, and only in patients in whom necrosis has already been excluded. The primary endpoint of that first-generation protocol is product-related safety through day 30, with day 90 as a sensitivity window: new or worsening bowel ischemia or new resection after enrollment; product-related bacteremia or invasive infection; aspiration, perforation, or barotrauma attributable to the delivery route; and product-related serious adverse events leading to discontinuation. Appropriate secondary endpoints are 30-day and 90-day all-cause mortality; delayed rescue laparotomy after enrollment; hospital and ICU length of stay among survivors; 30-day infection and unplanned readmission; early detection of administered strains or donor taxa; durable engraftment only when a pre-specified persistence window is met; and change in one pre-specified construct-specific research marker. Radiographic reduction of portal gas and time to complete resolution on protocolized imaging may be exploratory imaging endpoints. Need for laparotomy and survival remain outcomes of the standard surgical pathway, not efficacy targets of FMT or phage. Sample-size planning must not import THEMATIC’s *post hoc* HE-recurrence odds ratio or cirrhosis HVPG estimates as if they were HPVG effect sizes (83, 85). Operational definitions and the observational-versus-interventional hierarchy are summarized in Section 7.2.1 and **Table 4**.

3. Biomarker qualification. Prospective validation must treat each analyte as a distinct construct rather than as a pooled panel. I-FABP should be tested against necrosis and the decision to operate. Dual-sugar tests or future authentic pre-haptoglobin-2 assays, not commercial zonulin-family peptide kits, should be used if epithelial leak is the target. Commercial zonulin ELISAs should not enter an HPVG research composite, an emergency algorithm, or a claim of paracellular leakiness. LBP and soluble CD14 should be interpreted only as nonspecific PAMP-exposure readouts. Circulating PV-1, if measured at all, requires a stated research ELISA, a reported limit of detection, and paired portal–peripheral sampling before it can be treated as a GVB blood test. Breath hydrogen and methane, targeted gas-taxon quantification, and CT gas grading belong to a separate fermentation-and-burden arm. The aim is to test whether any of these research markers add information after ischemia laboratories and CT have already been used, not to replace those tools (69, 75).

4. Cellular and spatial resolution. Single-cell and spatial transcriptomic approaches should map the responses of LSECs, HSCs, Kupffer cells, and mucosal immune cells to microbial signals, with explicit comparison of acute ischemic versus chronic portal-hypertensive contexts.

5. Implementation science. Point-of-care barrier-immune assays suitable for the emergency department, and pragmatic trials embedded in acute-care services, will determine whether these immunological tools can be used without delaying surgical decisions.

Collectively these axes convert the physical-core plus optional-amplifier model from a conceptual synthesis into a set of experimentally actionable hypotheses.

#### 7.2.1 Defined clinical endpoints for future HPVG-focused studies

The six axes are not self-sufficient without a hierarchy of time-stamped clinical events. We therefore separate an all-comer observational registry (Study A) from a later necrosis-excluded interventional protocol (Study B). No microbiota-directed product is an HPVG therapy in either design. Proxy-trial numbers from cirrhosis, including hepatic venous pressure gradient change and the *post hoc* THEMATIC contrast in overt HE recurrence, are not HPVG effect sizes and are not used for sample-size planning.

Study A is a prospective multicenter observational registry of radiographically confirmed adult HPVG. Co-primary clinical endpoints are (i) all-cause in-hospital mortality, with 30-day and 90-day landmarks, and (ii) therapeutic source control within 72 hours of the index CT, defined as resection of histologically confirmed transmural necrosis or infarction. Secondary clinical endpoints are emergency surgical exploration within 24 hours of the index scan; operative decomposition into therapeutic resection, non-therapeutic or negative laparotomy, planned second look, delayed rescue laparotomy after an initial conservative pathway, and palliation without operation; hospital and intensive-care length of stay analysed among survivors and as time-to-discharge with death as a competing risk; new or progressive organ failure at 24–72 hours; 30-day culture-proven bacteremia or intra-abdominal infection; and 30-day unplanned readmission. Time to complete radiographic resolution of intrahepatic portal gas, and change in a pre-specified semi-quantitative computed-tomography grade on a stated reconstruction kernel and contrast phase, are imaging secondaries. Complete resolution is defined as disappearance of peripheral intrahepatic portal lucencies used to separate HPVG from pneumobilia. These imaging endpoints test visibility and washout. They are not surrogates for survival, because available series report median clearance of approximately four days among clinical responders and no consistent relationship between absorption time and outcome. Immune-microbiome assays remain tertiary research covariates. They may be tested for incremental association with the co-primary events after ischemia laboratories and CT have been used. They do not assign operative versus conservative care. Isolated iatrogenic or purely pressure-related cases remain in Study A as a natural-history comparator and are excluded *a priori* from Study B. Portal hypertension, when present, is recorded as a host covariate and is not a third phenotype label.

Study B is an interventional protocol that opens only after the observational safety register exists, and only after transmural necrosis and isolated iatrogenic or pressure-related entry have been excluded. The primary endpoint is protocol-defined product-related safety through day 30. Secondary clinical endpoints are 30-day and 90-day all-cause mortality, delayed rescue laparotomy or new resection after enrollment, length of stay among survivors, and 30-day infection or readmission. Secondary biological endpoints are early detection of administered strains or donor taxa, durable engraftment only if a persistence window is declared *a priori*, and change in one construct-specific research marker rather than a pooled panel. Change in computed-tomography gas grade and time to complete radiographic resolution are exploratory imaging endpoints confined to this eligibility window. Avoidance of laparotomy and all-cause mortality are not primary or co-primary efficacy targets of FMT, phage, or live probiotics. If a later confirmatory trial is ever considered, its primary endpoint would still need to be a patient-centered event in the necrosis-excluded window, such as 90-day all-cause death or a composite of death plus new ischemia plus new source-control failure, not gas clearance alone.

If validated, this framework could identify a subset of non-necrotic HPVG in which a later, protocolized microbiota-directed experiment is rational. Validation would not make those products current therapy.

### 7.3 Translational barriers and implementation constraints

Several obstacles separate this immunological framework from bedside use, and naming them defines the work ahead. Rarity and heterogeneity remain fundamental: HPVG is uncommon and etiologically diverse, so adequately powered, phenotype-homogeneous cohorts will require multicenter and likely international collaboration with harmonized case definitions. Sampling in the acute setting is constrained, because obtaining pre-intervention stool, mucosa, or blood for immune profiling from an acutely ill patient, who is often already receiving antibiotics, is logistically and ethically difficult. This biases available data toward stabilized or chronic states and confounds baseline microbiome and immune assessment. Causal inference is incomplete: association studies cannot establish that dysbiosis contributes to portal gas. This gap is bridgeable only with gnotobiotic and defined-consortium models engineered to test HPVG-relevant immune endpoints such as GVB failure, innate activation, portal-gas burden, and portal pressure, which do not yet exist as standardized systems. Intervention timing and safety remain decisive. The narrow therapeutic window in acute necrosis, and the PROPATRIA precedent, mean that trials must be designed with acuity stratification and rigorous safety monitoring from the outset (101). Finally, microbiome assays, FMT products, and barrier-immune biomarker platforms remain insufficiently standardized for regulatory-grade decision-making, although emerging consensus documents on FMT practice and on blood-based barrier biomarkers are beginning to close this gap (69, 85). The assay limits themselves are already specified in **Table 3**. Progress will depend less on new hypotheses than on the collaborative infrastructure needed to test the immunological propositions assembled here.

## 8 Conclusions

This review assembled evidence from cirrhosis, PSVD, intestinal ischemia/reperfusion, and primary mucosal inflammatory diseases to argue that gut dysbiosis is a biologically coherent candidate contributor to selected non-iatrogenic HPVG presentations through failure of mucosal and hepatic innate immunity. That argument is inferred from proxy systems. The distinctive claim is that intraportal gas is first the radiographic expression of a physical core, a route of entry plus a local driving gradient from lumen or wall into mesenteric veins, while a gas-generating community and epithelial or GVB immune failure are optional amplifiers of volume, duration, and inflammatory cost (Section 4.4). The established immediate accounts remain mechanical mucosal disruption with raised intraluminal pressure and/or local production and tracking of gas by cultured gas-forming bacteria, and these accounts can stand alone.

Three controversies follow. Directionality remains unresolved, because portal hypertension itself can generate a cirrhosis-like dysbiosis, because barrier-adjacent endotoxemia can precede a measurable portosystemic gradient in PSVD, and because antibiotics, intestinal ischemia, and critical illness can independently produce a similar community in acute HPVG. Dysbiosis is neither necessary nor sufficient for HPVG. Iatrogenic pressure-related HPVG, acute necrotic HPVG, and HPVG that occurs in a chronic portal-hypertensive host pose different immunological questions (Section 3.3), and only the first of these is already explained by the physical core without a microbial requirement. In the third setting portal hypertension remains a host covariate rather than an upstream dysbiosis-driven source of intraportal gas. A fourth, definitional point is not controversial in this review: portal vein thrombosis, portal hypertension, pneumatosis intestinalis, pyogenic liver abscess, and HPVG are not interchangeable vascular or infectious manifestations.

No microbiota-directed product has been tested against an HPVG endpoint. THEMATIC strengthens the case for studying microbiome restoration in cirrhosis with HE; it does not authorize treatment of HPVG. PROPATRIA remains a standing caution against unselected live-microbe administration in the acute abdomen (Section 6.5). Realizing a testable research program will require necrosis-stratified longitudinal cohorts, colonization models that quantify intraportal gas, and, only after an observational safety step, interventional studies whose first endpoints are safety and biological response rather than avoidance of laparotomy. Collaboration among emergency physicians, hepatologists, radiologists, microbiologists, and surgeons will be required to assemble the rare cases needed for adequately powered study and to keep microbiome science inside, not against, time-critical surgical decision-making.
